# High-Precision computational solutions for nonlinear evolution models in graphene sheets

**DOI:** 10.1038/s41598-025-85263-0

**Published:** 2025-02-01

**Authors:** Mostafa M. A. Khater, Suleman H. Alfalqi, Aleksander Vokhmintsev

**Affiliations:** 1 School of Medical Informatics and Engineering, Xuzhou Medical University, 209 Tongshan Road, 221004 Xuzhou, Jiangsu Province P. R. China; 2 Institute of Digital Economy, Ugra State University, Khanty-Mansiysk, 628012 Russia; 3 Department of Basic Science, The Higher Institute for Engineering & Technology, Al-Obour, Cairo 10587 Egypt; 4 Department of Mathematics, Faculty of Science and Arts in Mahayil Asir, King Khalid University, Abha, Saudi Arabia; 5 Institute of Information Technology, Chelyabinsk State University, Chelyabinsk, Russia

**Keywords:** Graphene sheets model, Khater methods, Generalized rational method, Variational iteration method, Applied mathematics, Computational science

## Abstract

This study investigates the analytical solutions of a nonlinear evolution model governing the dynamics of graphene sheets, a material renowned for its exceptional electronic properties and versatile applications in nanotechnology. Three advanced analytical approaches-the Khater II (Khat II) method, the Khater III (Khat III) method, and the Generalized Rational (GRat) approach-are employed to derive exact solutions for this model with high precision. The accuracy and reliability of these solutions are validated by comparing them to numerical results obtained via He’s Variational Iteration (HVI) method, which serves as a benchmark for numerical verification. The analysis reveals a remarkable agreement between the analytical and numerical solutions, highlighting the robustness and effectiveness of the proposed methodologies. Furthermore, this study provides new insights into the nonlinear dynamics and physical properties of graphene sheets, while also identifying connections to other prominent nonlinear evolution equations. The innovative use of these analytical techniques offers practical frameworks for addressing complex nonlinear models in mathematical physics, thus advancing solution methodologies for such equations. This research contributes significantly to applied mathematics, material science, and nanotechnology by delivering accurate solutions and enhancing our understanding of graphene’s nonlinear behavior. Finally, the findings have far-reaching implications, offering potential applications in designing advanced materials with tailored properties to support technological advancements, thereby pushing the boundaries of nanotechnology and materials engineering.

## Literature review

Graphene, a single-layer two-dimensional material composed of carbon atoms arranged in a hexagonal lattice, has revolutionized material science due to its unparalleled electronic, thermal, and mechanical properties^1–8^. The study of graphene’s behavior frequently necessitates the use of complex nonlinear evolution equations, which are critical for modeling phenomena such as electronic transport, mechanical deformation, and thermal conductivity within graphene sheets^9^. Despite considerable advancements in the field, solving these equations analytically remains a significant challenge^10^.

The (2+1)-dimensional graphene sheets (GS) model, which captures the behavior of graphene under diverse conditions, has yet to be fully addressed through analytical solutions^11^. This absence of comprehensive analytical solutions restricts our ability to gain deeper insights into graphene’s distinctive properties and behaviors^12^. Bridging this gap is essential for advancing both the theoretical foundations and practical applications of material science and nanotechnology^13^.

Previous research has extensively investigated the properties and behaviors of graphene. Yoon and Young Ki (2008) emphasized the groundbreaking discovery of graphene and its fundamental characteristics^14^. Neto et al. (2009) provided a detailed review of its electronic properties, highlighting its potential for various applications^15^. Recent studies have explored methods such as the Hirota bilinear method and the Adomian decomposition method to solve nonlinear evolution equations associated with graphene^16–20^. However, these approaches often encounter limitations in terms of accuracy and generalizability, highlighting the need for more robust analytical methods.

The primary aim of this study is to solve the (2+1)-dimensional graphene sheets (GS) model using advanced analytical approaches, including the Khater II (Khat II), Khater III (Khat III), and Generalized Rational (GRat) methods. Additionally, the solutions obtained through these methods will be validated using He’s Variational Iteration (HVI) method, which provides a reliable numerical benchmark to assess the accuracy and effectiveness of the proposed analytical solutions.

This research introduces innovative analytical methodologies that significantly enhance our understanding of the complex behavior of graphene, a material of critical importance in contemporary material science. Consequently, this work makes substantial contributions to applied mathematics and material science, offering reliable solutions that have the potential to advance the development of tailored materials for diverse technological applications.

It is important to note that this study focuses on the (2+1)-dimensional model describing the behavior of graphene sheets (GS). This model operates under the idealized assumption of unperturbed dynamics, excluding external influences. The scope of this research is inherently limited by the specific analytical methods employed and the numerical validation techniques utilized. Therefore, the results presented may not be directly applicable to higher-dimensional models or more complex scenarios that aim to account for additional factors affecting graphene’s behavior.

The structure of the paper is organized as follows: Section 2 explains the investigated model’s mathematical structure. Section 3 studies the dynamical behavior of the nonlinear investigated model by employing the bifurcation analysis to explain the model’s equilibrium points through the Hamiltonian function. Section 4 evaluates the accuracy of the derived solutions and presents several novel solitary wave solutions relevant to the investigated model. This section also includes a comprehensive stability analysis, supported by graphical representations. Section 5 provides a detailed discussion of the solutions’ accuracy and their broader scientific significance. It emphasizes the contributions of the study within the context of ongoing research in the field. Section 6 gives a demonstration of the constructed solutions and the whole study’s results. Section 7 summarizes the key findings and overarching conclusions, encapsulating the primary aspects of this investigation.

## Investigated model’s mathematical structure

The (2+1)-dimensional GS model is mathematically given by^21,22^

1 $$\begin{aligned} \frac{\partial ^2\mathcal {B}}{\partial x\, \partial t}+\frac{\partial }{\partial x}\left( \frac{\partial ^3\mathcal {B}}{\partial x^{3}}+\rho _1 \,\frac{\partial \mathcal {B}}{\partial x}+\mathcal {B} \,\frac{\partial \mathcal {B}}{\partial x}\right) +\rho _2 \,\frac{\partial ^2\mathcal {B}}{\partial y^{2}}=0, \end{aligned}$$

where the function $\mathcal {B}(x, y, t)$represents the amplitude of the wave function in the graphene sheet^23^. This wave function is influenced by both the spatial coordinates $x$ and $y$, and the temporal coordinate $t$. While the physical interpretation of each term and parameter can be explained as following^24,25^:

- i) **Terms:**
  - $\bullet$ $\frac{\partial ^2 \mathcal {B}}{\partial x \, \partial t}$: This term represents the mixed partial derivative of the wave function with respect to both the spatial coordinate $x$ and the temporal coordinate $t$. It indicates the rate of change of the slope of $\mathcal {B}$ in the $x$ direction over time.
  - $$\bullet$$ $$\frac{\partial }{\partial x}\left( \frac{\partial ^3 \mathcal {B}}{\partial x^{3}} + \rho _1 \,\frac{\partial \mathcal {B}}{\partial x} + \mathcal {B} \,\frac{\partial \mathcal {B}}{\partial x}\right)$$:
    - a) $\frac{\partial }{\partial x}\left( \frac{\partial ^3 \mathcal {B}}{\partial x^{3}}\right)$: This represents the spatial dispersion term, which is the third-order spatial derivative of the wave function with respect to $x$, indicating the nonlinearity and dispersion effects in the $x$ direction.
    - b) $\frac{\partial }{\partial x}\left( \rho _1 \,\frac{\partial \mathcal {B}}{\partial x}\right)$: This term incorporates $\rho _1$, a parameter related to the linear wave propagation along the $x$ direction. It indicates linear attenuation or amplification along $x$.
    - c) $\frac{\partial }{\partial x}\left( \mathcal {B} \,\frac{\partial \mathcal {B}}{\partial x}\right)$: This is the nonlinear convection term, representing self-interaction of the wave function $\mathcal {B}$ along $x$.
  - $\bullet$ $\rho _2 \,\frac{\partial ^2 \mathcal {B}}{\partial y^{2}}$: This term accounts for the diffusion or dispersion of the wave function in the transverse spatial direction $y$. The parameter $\rho _2$ is related to the rate of this diffusion or dispersion.
- ii) **Parameters:**
  - $\bullet$ $\rho _1$: This parameter is related to the linear propagation characteristics of the wave function in the $x$ direction. It can represent material properties or external conditions affecting wave propagation in that direction.
  - $\bullet$ $\rho _2$: This parameter governs the transverse diffusion or dispersion effects in the $y$ direction. It reflects the influence of transverse spatial variations on the behavior of the wave function.

In summary, the given equation (Eq. (1)) models the behavior of wave functions in a (2+1) dimensional graphene sheet^26^. The wave function $\mathcal {B}(x, y, t)$ evolves according to a combination of nonlinear and linear propagation terms in the $x$ direction, and a transverse diffusion term in the $y$ direction. The parameters $\rho _1$ and $\rho _2$modulate these effects, reflecting the material and physical properties of the graphene sheet^27^.

In this context, we implement $\mathcal {B}(x, y, t)=\psi (\mathfrak {T}),\, \mathfrak {T}=c \,t+\eta _1 \,x+\eta _2 \,y$, where $c,\, \eta _1,\, \eta _2$ can be explained as follows:

- $c$: This parameter represents the wave speed. It determines how fast the wave propagates in time. A larger $c$ indicates a faster wave, while a smaller $c$ indicates a slower wave. In the context of graphene, this speed can be influenced by the electronic properties and the interactions within the material. This is a measure of how quickly the wave travels through the graphene sheet. It can be influenced by various factors, including the intrinsic properties of graphene and external conditions such as temperature or applied fields.
- $\eta _1$: This parameter is the wave number component in the $x$ direction. It describes how the phase of the wave changes with respect to the $x$ coordinate. The value of $\eta _1$ determines the spatial frequency of the wave in the $x$ direction, affecting the wavelength and the direction of wave propagation. A higher $\eta _1$ corresponds to shorter wavelengths and higher spatial frequencies in the $x$ direction. This quantifies the spatial oscillations of the wave along the $x$ axis. It is related to the periodicity and propagation direction of the wave component in the $x$ direction.
- $\eta _2$: This parameter is the wave number component in the $y$ direction. Similar to $\eta _1$, $\eta _2$ describes how the phase of the wave changes with respect to the $y$ coordinate. It affects the spatial frequency of the wave in the $y$ direction, influencing the wavelength and the direction of wave propagation in the transverse direction. A higher $\eta _2$ corresponds to shorter wavelengths and higher spatial frequencies in the $y$ direction. This quantifies the spatial oscillations of the wave along the $y$ axis. It is related to the periodicity and propagation direction of the wave component in the $y$ direction.

The wave transformation $\mathfrak {T} = c \, t + \eta _1 \, x + \eta _2 \, y$ essentially describes a moving wavefront in the graphene sheet with a specific speed and direction. The direction and nature of the wave propagation are determined by the relative magnitudes and signs of $c$, $\eta _1$, and $\eta _2$. This transformation simplifies the analysis by converting the partial differential equation into a simpler form, often reducing it to an ordinary differential equation in terms of $\mathfrak {T}$. This simplification aids in finding analytical or numerical solutions to the original problem., to convert Eq. (1) into the next ordinary differential equation

2 $$\begin{aligned} c\, \eta _1\, \psi ''+\eta _2^2 \,\rho _2 \,\psi ''+\eta _1^2 \,\left( \left( \rho _1+\psi \right) \, \psi ''+\left( \psi '\right) ^2\right) +\eta _1^4 \,\psi ^{(4)}=0. \end{aligned}$$

Twice integration of Eq. (2) with respect to $\mathfrak {T}$ along with zero integration constant, yields

3 $$\begin{aligned} \psi \left( c \,\eta _1+\eta _1^2 \,\rho _1+\eta _2^2 \,\rho _2\right) +\eta _1^4 \,\psi ''+\frac{1}{2} \,\eta _1^2 \,\psi ^2=0. \end{aligned}$$

Balancing the terms of Eq. (3) by using the homogeneous balance rule along with the suggested analytical schemes’ auxiliary equations

$$\begin{aligned} {\left\{ \begin{array}{ll} f'(\mathfrak {T} )^2\rightarrow \frac{1}{\ln ^2(K)} \left( \alpha +K^{f(\mathfrak {T} )} \left( \beta +\sigma \, K^{f(\mathfrak {T} )}\right) \right) , & \text {Khat III~method,}\\ \\ f'(\mathfrak {T} )\rightarrow -\delta -f(\mathfrak {T} )^2,\phi '(\mathfrak {T} )\rightarrow -f(\mathfrak {T} )\, \phi (\mathfrak {T} ), & \text {Khat II~ method,}\\ \\ \phi '(\mathfrak {T} )\rightarrow \zeta +\varrho \, \phi (\mathfrak {T} )^2, & \text {GRat~method}, \end{array}\right. } \end{aligned}$$

where $\alpha ,\, \beta ,\, \sigma ,\, \delta ,\, \zeta ,\, \varrho$ are arbitrary constants to be determined later., leads to construct the general solutions of the investigated model in the next form

4 $$\begin{aligned} \psi (\mathfrak {T})= {\left\{ \begin{array}{ll} \sum \limits _{i=0}^{2 \,m} a_i \left( K^{f(\mathfrak {T} )}\right) ^i, & \text {Khat III~method,}\\ \\ \sum \limits _{i=1}^n \left( a_i \,f(\mathfrak {T} )^i+b_i \,\phi (\mathfrak {T} ) f(\mathfrak {T} )^{i-1}\right) +a_0, & \text {Khat II~ method,}\\ \\ \sum \limits _{i=0}^n a_i \left( \frac{\psi '(\mathfrak {T} )}{\psi ^2(\mathfrak {T} )}\right) ^i, & \text {GRat~method}, \end{array}\right. } \end{aligned}$$

where $a_{i},\, b_{i}$ are arbitrary constants to be determined through the employed analytical techniques’ headlines.

##  (2+1)–Dimensional GS Model’s Dynamics

This study employs bifurcation analysis to investigate the dynamical behaviour of the nonlinear GS model, including the elucidation of equilibrium points, the exploration of chaotic and quasi-periodic behaviours, and the illustration of the planar dynamical system’s sensitive nature.

### Bifurcation analysis

This section analyses the possible phase graphs of the planar dynamical system after it is turned into a dynamical system via the Galilean transformation, as specified by Eq. (3).

5 $$\begin{aligned} \left\{ \begin{aligned}&\frac{d\,\psi }{d\, \mathfrak {Z}}=\upsilon ,\\ \\&\frac{d\,\upsilon }{d\, \mathfrak {Z}}=-\frac{\psi \left( 2 c \eta _1+\eta _1^2 \left( 2 \rho _1+\psi \right) +2 \eta _2^2 \rho _2\right) }{2 \eta _1^4}. \end{aligned} \right. \end{aligned}$$

The phase orbits of (5) provide the exact traveling wave solutions of (1), grounded on planar dynamical theory. In this setting, the Hamiltonian function $\mathcal {H}(\psi ,\,\upsilon )$ may be articulated in the following manner

6 $$\begin{aligned} \mathcal {H}(\psi ,\,\upsilon )=\frac{3\, c\, \eta _1\, \psi ^2+\eta _1^2\, \psi ^2 \,\left( 3\, \rho _1+\psi \right) +3\, \eta _2^2 \,\rho _2\, \psi ^2+3\, \eta _1^4\, \upsilon ^2}{6\, \eta _1^4}=h_0, \end{aligned}$$

where $\left( \frac{\upsilon ^2}{2}\right)$ represents the kinetic energy, $\left( \frac{3\, c\, \eta _1\, \psi ^2+\eta _1^2\, \psi ^2 \,\left( 3\, \rho _1+\psi \right) +3\, \eta _2^2 \,\rho _2 \,\psi ^2}{6\, \eta _1^4}\right)$ represents the potential energy, and $h_0$ represents the total energy.

Deriving the equilibrium points of system (5), leads to

7 $$\begin{aligned} \left\{ \begin{aligned}&\frac{d\,\psi }{d\, \mathfrak {Z}}=\upsilon =0,\\ \\&\frac{d\,\upsilon }{d\, \mathfrak {Z}}=-\frac{\psi \left( 2 c \eta _1+\eta _1^2 \left( 2 \rho _1+\psi \right) +2 \eta _2^2 \rho _2\right) }{2 \eta _1^4}=0. \end{aligned} \right. \end{aligned}$$

Thus, we get the equilibrium points is given by $E_{1}=\left( 0,0\right) ,\, E_{2}=-\frac{2 \left( \eta _1 \left( c+\eta _1 \rho _1\right) +\eta _2^2 \rho _2\right) }{\eta _1^2}$. The Jacobian matrix $J (\psi ,\,\upsilon )$ of the following Hamiltonian system at an equilibrium point $\left( \psi _{0},\,\upsilon _{0}\right)$

8 $$\begin{aligned} \mathcal {J} (\psi , \upsilon )=\left( \begin{array}{cc} 0 & 1 \\ -\frac{\eta _1 \left( c+\eta _1 \left( \rho _1+u\right) \right) +\eta _2^2 \rho _2}{\eta _1^4}& 0 \\ \end{array} \right) \end{aligned}$$

and its determinant is given by

9 $$\begin{aligned} |\mathcal {J} (\psi , \upsilon )|=\frac{\eta _1 \left( c+\eta _1 \left( \rho _1+u\right) \right) +\eta _2^2 \rho _2}{\eta _1^4}. \end{aligned}$$

Theorem formulation is feasible through the application of bifurcation theory to planar dynamical systems.

#### Theorem 3.1

Let $\mathcal {J}\left( \mathcal {E}\left( \psi _0, \upsilon _0\right) \right)$ denote the coefficient matrix of the linearized planar dynamic system at the equilibrium point $\mathcal {E}\left( \psi _0, \upsilon _0\right)$, and let $\left| \mathcal {J}\left( \mathcal {E}\left( \psi _0, \upsilon _0\right) \right) \right|$ represent its determinant (the Jacobian determinant). The nature of the equilibrium point is determined as follows:

- If $\left| \mathcal {J}\left( \mathcal {E}\left( \psi _0, \upsilon _0\right) \right) \right| < 0$, the equilibrium point is a **saddle point**.
- If $\left| \mathcal {J}\left( \mathcal {E}\left( \psi _0, \upsilon _0\right) \right) \right|> 0$ and $\operatorname {Trace}\left( \mathcal {J}\left( \mathcal {E}\left( \psi _0, \upsilon _0\right) \right) \right) = 0$, the equilibrium point is classified as a **center**.
- If $\left| \mathcal {J}\left( \mathcal {E}\left( \psi _0, \upsilon _0\right) \right) \right|> 0$ and $\operatorname {Trace}\left( \mathcal {J}\left( \mathcal {E}\left( \psi _0, \upsilon _0\right) \right) \right) ^2 - 4\left| \mathcal {J}\left( \mathcal {E}\left( \psi _0, \upsilon _0\right) \right) \right| = 0$, the equilibrium point is identified as a **node**.
- If $\left| \mathcal {J}\left( \mathcal {E}\left( \psi _0, \upsilon _0\right) \right) \right| = 0$ and the Poincaré index of the equilibrium point equals zero, the point is referred to as a **cusp point**.

Currently, we are evaluating all potential scenarios in which the dynamical system (5) undergoes bifurcation. The following conclusion is based on a qualitative analysis of the facts:

**Table 1:** Equilibrium points (Center ($\mathcal {C}$) and Saddle ($\mathcal {S}$)) for the investigated model based on a different values of the above-mentioned parameters.

**Case**	$\eta _1$	$\eta _2$	$\rho _1$	$\rho _2$	$c$	$\mathcal {E}_{1}$	$\mathcal {E}_{2}$
i	$0.5$	$0.667$	$0.75$	$0.8$	$0.833$	$\mathcal {C}$	$\mathcal {S}$
ii	$0.5$	$0.667$	$0.75$	$0.8$	$-0.833$	$\mathcal {C}$	$\mathcal {S}$
iii	$0.5$	$0.667$	$0.75$	$-0.8$	$0.833$	$\mathcal {C}$	$\mathcal {S}$
iv	$0.5$	$0.667$	$0.75$	$-0.8$	$-0.833$	$\mathcal {S}$	$\mathcal {C}$
v	$0.5$	$0.667$	$-0.75$	$0.8$	$0.833$	$\mathcal {C}$	$\mathcal {S}$
vi	$0.5$	$0.667$	$-0.75$	$0.8$	$-0.833$	$\mathcal {S}$	$\mathcal {C}$
vii	$0.5$	$0.667$	$-0.75$	$-0.8$	$0.833$	$\mathcal {S}$	$\mathcal {C}$
viii	$0.5$	$0.667$	$-0.75$	$-0.8$	$-0.833$	$\mathcal {S}$	$\mathcal {C}$
ix	$0.5$	$-0.667$	$0.75$	$0.8$	$0.833$	$\mathcal {C}$	$\mathcal {S}$
x	$0.5$	$-0.667$	$0.75$	$0.8$	$-0.833$	$\mathcal {C}$	$\mathcal {S}$

**Fig. 1:**
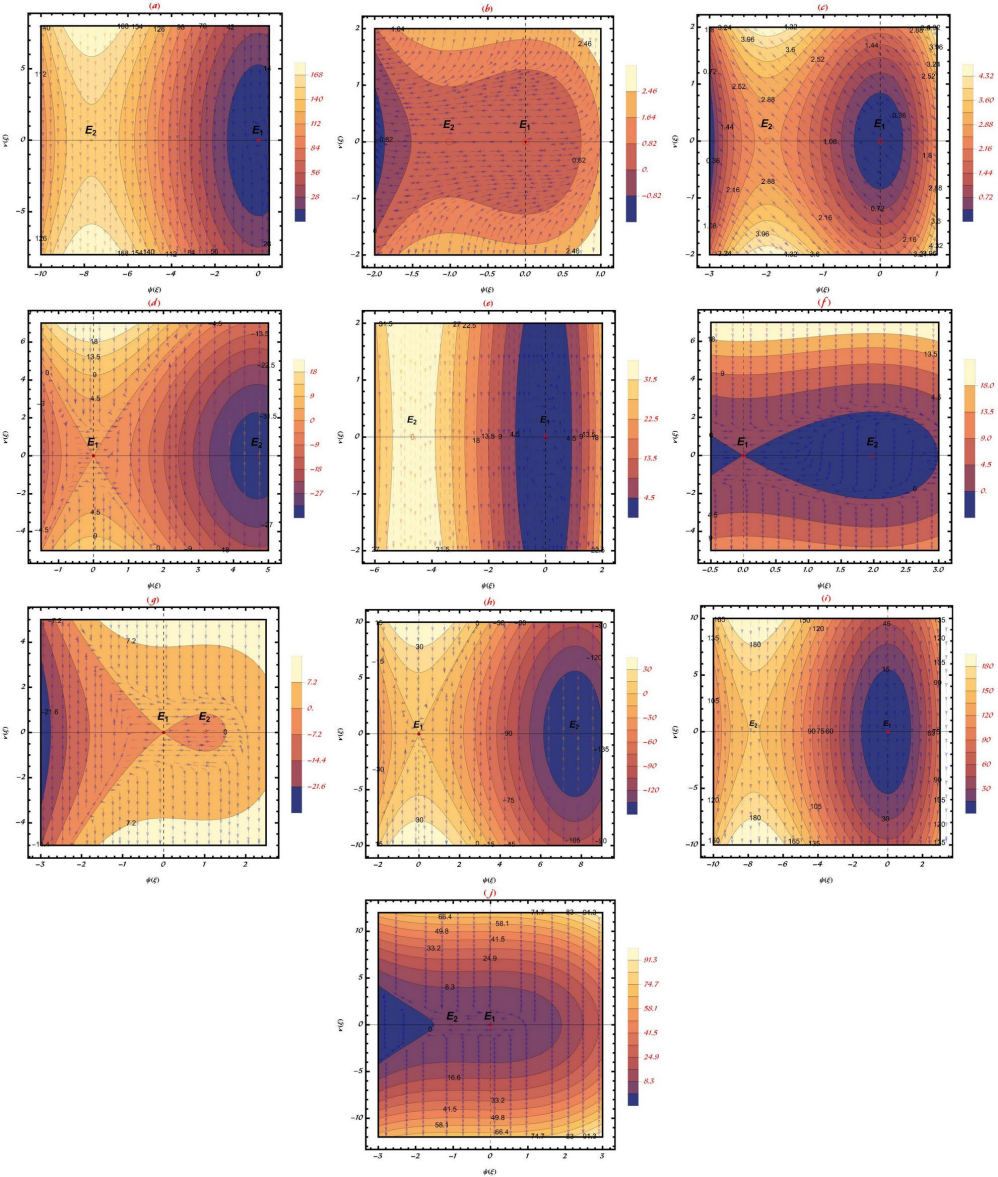
Bifurcation diagram of analysed graphene system phase pictures. Parameter values define equilibrium points, which might be stable or unstable. Transitions between states at bifurcation points reveal system stability and solution properties.

### Chaotic analysis and quasi-periodic behaviors

Periodic disruptions are common in the physical settings of the actual world, and they have the potential to significantly negatively impact some systems. Anarchy is likely to emerge as a consequence of this circumstance. In order to determine if the system (5) displays chaotic behaviour when subjected to the impact of a perturbation term, we will conduct an investigation. The perturbation term $f_{0} \,cos (\mu \,\mathfrak {Z})$ is integrated in order to provide clarity about the model that has been highlighted. As a result, the modified term makes adjustments to the two-dimensional system that is shown in Eq. (5)

10 $$\begin{aligned} \left\{ \begin{aligned}&\frac{d\,\psi }{d\, \mathfrak {Z}}=\upsilon ,\\ \\&\frac{d\,\upsilon }{d\, \mathfrak {Z}}=\frac{\psi ^3}{3}-\psi \, \left( -3 \zeta _1^2+\zeta _1+\zeta _2+\kappa \right) +f_{0} \,cos (\mu \,\eta ) . \end{aligned} \right. \end{aligned}$$

The process’s amplitude is represented by the symbol $f_{0}$. The frequency of the system in question is represented by $\mu$. Minor perturbations in chaotic systems can have a significant impact on nonlinear systems, leading to behaviours that are both quasi-periodic and chaotic. Utilising the system equation (10), we conducted an examination of the chaotic dynamics. The alteration in frequency is the source of quasi-periodic waves. The system dynamics described by (10) are examined in this section by modifying the amplitude $f_{0}$ and frequency $\mu$.

**Fig. 2:**
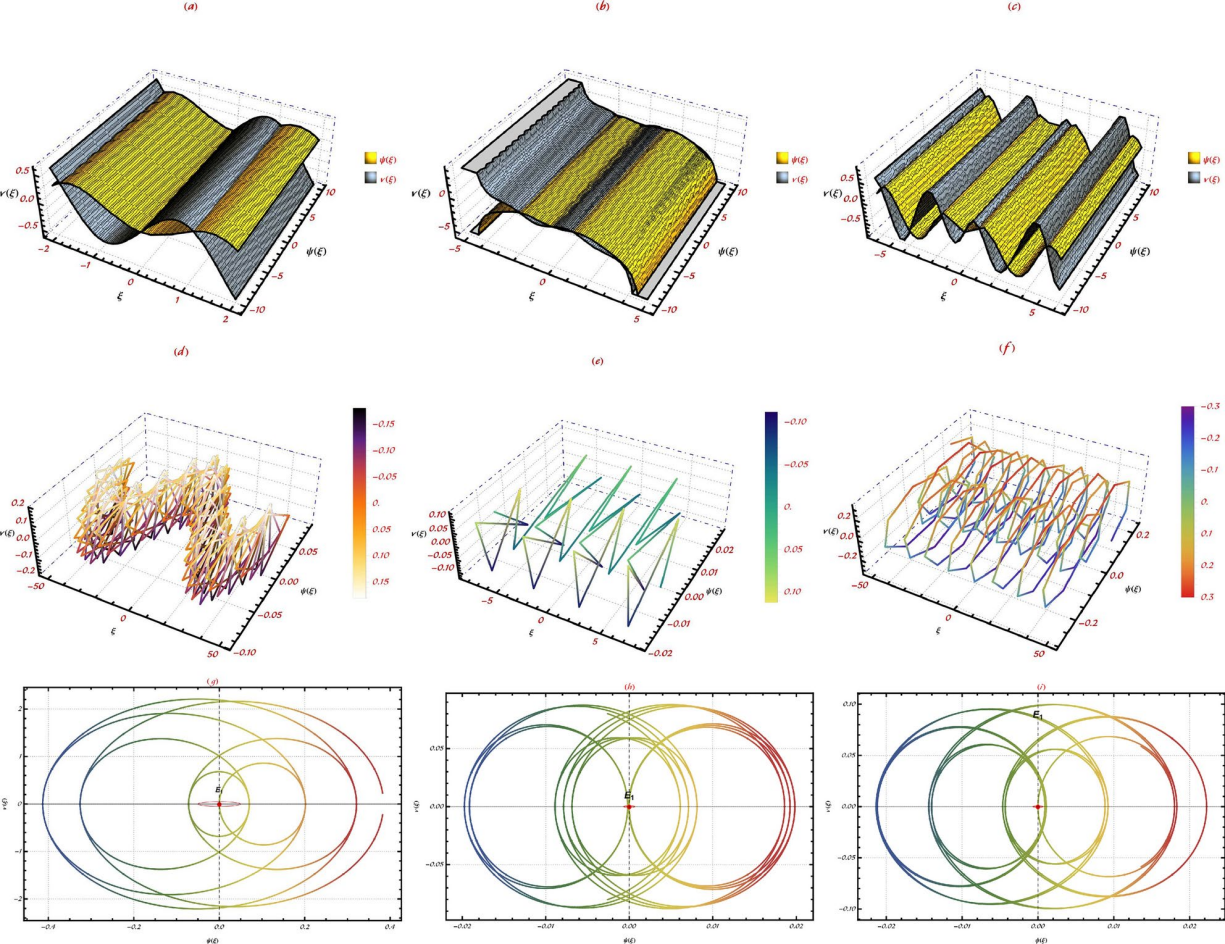
Phase visuals of the nonlinear dynamical system (10), illustrating the system’s behavior under various conditions. The figure highlights the influence of amplitude ($\mu$ ) and frequency ($f_{0}$) on the dynamics, showing the system’s progression through state space and emphasizing regions of stability and chaos.

### Sensitivity assessment

**Fig. 3:**
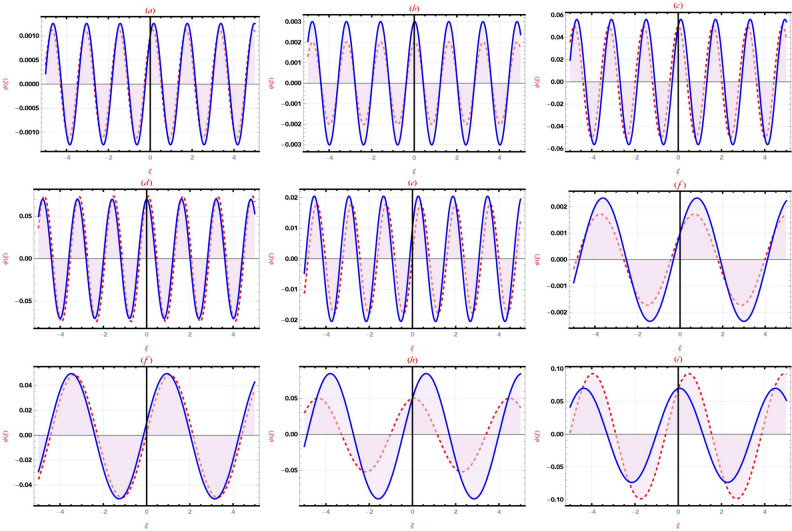
Visualizations of sensitivity in the nonlinear dynamical system (10), demonstrating how beginning circumstances affect its behavior. This graphic illustrates how little changes in beginning values significantly alter wave forms ($\left( \psi (\mathfrak {Z}), \upsilon (\mathfrak {Z})\right)$. The sensitivity analysis shows the model’s responsiveness to beginning circumstances, emphasizing the need of proper parameter selection in system dynamics prediction.

Here, we examine the sensitivity characteristics of the planar dynamical system delineated by the equation (5). The objective of this endeavour is to assess the sensitivity shown by the chosen model now under examination. For the sensitivity analysis, the parametric values of $\left( \kappa \rightarrow 1,\,\zeta _1\rightarrow 0.5,\,\zeta _2\rightarrow 1\right)$ are used. As seen in Figure 3, even little modifications to these essential factors substantially affect the model’s performance. This illustrates the structure’s susceptibility to the circumstances to which it was originally subjected.

**Fig. 4:**
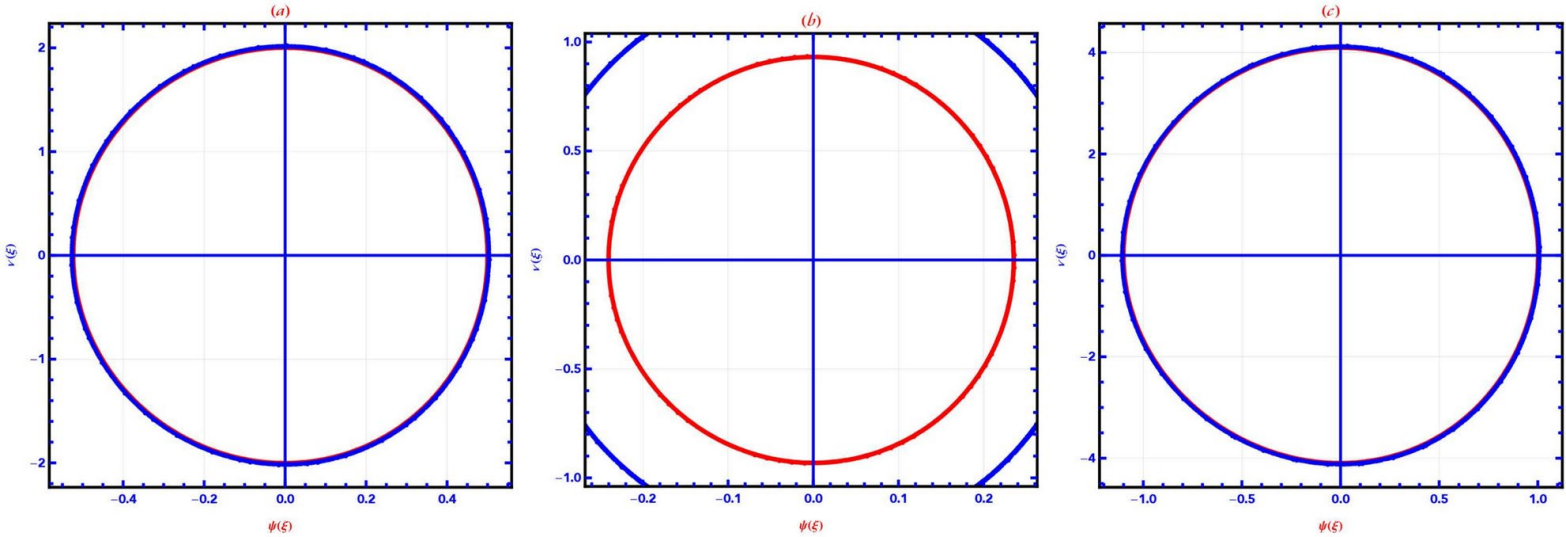
The sensitivity research of the nonlinear dynamical system (10) involves examining effects from various beginning positions. Comparing wave-forms $(\left( \psi (\mathfrak {Z}), \upsilon (\mathfrak {Z})\right)$ reveals the tremendous influence of small changes in initial values on system dynamics The findings show the complicated connection between factors and system responses and how beginning circumstances affect HM system performance.

## Accurate and novel solitary wave solutions

In this section, the Khat III, II and GRat methods are implemented to construct novel solitary wave solutions for the (2+1) dimensional GS model. Additionally, the accuracy of the solutions is examined using the HVI scheme.

### Novel solitary wave solutions

Here, the suggested computational techniques are implemented to discover novel solitary wave solutions for the (2+1) dimensional GS model.

1. **Soliton wave via the Khat III method:**
  Evaluating the value of the above-mentioned parameters through the Khat III method yields
  **Set I**
  $$\begin{aligned} a_0\rightarrow 0,c\rightarrow \frac{a_1^2 \eta _1}{12 a_2}-\eta _1 \rho _1-\frac{\eta _2^2 \rho _2}{\eta _1},\alpha \rightarrow -\frac{a_1^2}{12 a_2 \eta _1^2},\beta \rightarrow -\frac{a_1}{6 \eta _1^2},\sigma \rightarrow -\frac{a_2}{12 \eta _1^2}. \end{aligned}$$
  **Set II**
  $$\begin{aligned} a_0\rightarrow \frac{a_1^2}{6 a_2},c\rightarrow -\frac{a_1^2 \eta _1}{12 a_2}-\eta _1 \rho _1-\frac{\eta _2^2 \rho _2}{\eta _1},\alpha \rightarrow -\frac{a_1^2}{12 a_2 \eta _1^2},\beta \rightarrow -\frac{a_1}{6 \eta _1^2},\sigma \rightarrow -\frac{a_2}{12 \eta _1^2}. \end{aligned}$$
  **Set III**
  $$\begin{aligned} a_1\rightarrow 0,c\rightarrow -\frac{1}{2} \eta _1 \left( a_0+2 \rho _1\right) -\frac{\eta _2^2 \rho _2}{\eta _1},\alpha \rightarrow -\frac{a_0}{8 \eta _1^2},\beta \rightarrow 0,\sigma \rightarrow -\frac{a_2}{12 \eta _1^2}. \end{aligned}$$
  **Set IV**
  $$\begin{aligned} a_0\rightarrow 0,a_1\rightarrow 0,c\rightarrow -\frac{4 \alpha \eta _1^4+\eta _1^2 \rho _1+\eta _2^2 \rho _2}{\eta _1},\beta \rightarrow 0,\sigma \rightarrow -\frac{a_2}{12 \eta _1^2}. \end{aligned}$$
  Thus, the solitary wave solutions of the investigated model are constructed by
  11 $$\begin{aligned} \mathcal {B}_{\text {I}}(x,y,t)= & -\frac{a_1^2 }{4 a_2} \sec ^2\left( \frac{a_1 \left( \eta _1 \left( \frac{a_1^2 t}{12 a_2}-\rho _1 t+x\right) -\frac{\eta _2^2 \rho _2 t}{\eta _1}+\eta _2 y\right) }{4 \sqrt{3} \sqrt{a_2} \eta _1}\right) , \end{aligned}$$
  12 $$\begin{aligned} \mathcal {B}_{\text {II}}(x,y,t)= & \frac{a_1^2 }{12 a_2} \left( 2-3 \sec ^2\left( \frac{a_1 \left( \eta _1 \left( -\frac{a_1^2 t}{12 a_2}-\rho _1 t+x\right) -\frac{\eta _2^2 \rho _2 t}{\eta _1}+\eta _2 y\right) }{4 \sqrt{3} \sqrt{a_2} \eta _1}\right) \right) , \end{aligned}$$
  13 $$\begin{aligned} \mathcal {B}_{\text {III}}(x,y,t)= & \frac{1}{2} a_0 \left( 2-3 \csc ^2\left( \frac{\sqrt{a_0} \left( \eta _1 \left( -\frac{a_0 t}{2}-\rho _1 t+x\right) -\frac{\eta _2^2 \rho _2 t}{\eta _1}+\eta _2 y\right) }{2 \sqrt{2} \eta _1}\right) \right) , \end{aligned}$$
  14 $$\begin{aligned} \mathcal {B}_{\text {IV}}(x,y,t)= & -12 \alpha \eta _1^2 \text {csch}^2\left( \sqrt{\alpha } \left( -\frac{\eta _2^2 \rho _2 t}{\eta _1}+\eta _1 \left( x-t \left( 4 \alpha \eta _1^2+\rho _1\right) \right) +\eta _2 y\right) \right) . \end{aligned}$$
2. **Soliton wave via the Khat II method:**
  Evaluating the value of the above–mentioned parameters through the Khat II method, gets
  **Set I**
  $$\begin{aligned} a_0\rightarrow -12 \delta \eta _1^2,a_1\rightarrow 0,a_2\rightarrow -12 \eta _1^2,b_1\rightarrow 0,b_2\rightarrow 0,c\rightarrow \frac{4 \delta \eta _1^4-\eta _1^2 \rho _1-\eta _2^2 \rho _2}{\eta _1}. \end{aligned}$$
  **Set II**
  $$\begin{aligned} a_0\rightarrow -4 \delta \eta _1^2,a_1\rightarrow 0,a_2\rightarrow -12 \eta _1^2,b_1\rightarrow 0,b_2\rightarrow 0,c\rightarrow \frac{-4 \delta \eta _1^4-\eta _1^2 \rho _1-\eta _2^2 \rho _2}{\eta _1}. \end{aligned}$$
  **Set III**
  $$\begin{aligned} a_0\rightarrow -4 \delta \eta _1^2,a_1\rightarrow 0,a_2\rightarrow -6 \eta _1^2,b_1\rightarrow 0,b_2\rightarrow 6 \sqrt{\delta } \eta _1^2,c\rightarrow \frac{-\delta \eta _1^4-\eta _1^2 \rho _1-\eta _2^2 \rho _2}{\eta _1}. \end{aligned}$$
  **Set IV**
  $$\begin{aligned} a_0\rightarrow -6 \delta \eta _1^2,a_1\rightarrow 0,a_2\rightarrow -6 \eta _1^2,b_1\rightarrow 0,b_2\rightarrow 6 \sqrt{\delta } \eta _1^2,c\rightarrow \frac{\delta \eta _1^4-\eta _1^2 \rho _1-\eta _2^2 \rho _2}{\eta _1}. \end{aligned}$$
  Thus, the solitary wave solutions of the investigated model are constructed by
  15 $$\begin{aligned} \mathcal {B}_{\text {I}}(x,y,t)= & -12 \delta \eta _1^2 \sec ^2\left( \sqrt{\delta } \left( 4 \delta \eta _1^3 t-\frac{\eta _2^2 \rho _2 t}{\eta _1}+\eta _1 \left( x-\rho _1 t\right) +\eta _2 y\right) \right) , \end{aligned}$$
  16 $$\begin{aligned} \mathcal {B}_{\text {I}}(x,y,t)= & -12 \delta \eta _1^2 \csc ^2\left( \sqrt{\delta } \left( 4 \delta \eta _1^3 t-\frac{\eta _2^2 \rho _2 t}{\eta _1}+\eta _1 \left( x-\rho _1 t\right) +\eta _2 y\right) \right) , \end{aligned}$$
  17 $$\begin{aligned} \mathcal {B}_{\text {II}}(x,y,t)= & -4 \delta \eta _1^2 \left( 3 \tan ^2\left( \sqrt{\delta } \left( -\frac{\eta _2^2 \rho _2 t}{\eta _1}+\eta _1 \left( x-t \left( 4 \delta \eta _1^2+\rho _1\right) \right) +\eta _2 y\right) \right) +1\right) , \end{aligned}$$
  18 $$\begin{aligned} \mathcal {B}_{\text {II}}(x,y,t)= & -4 \delta \eta _1^2 \left( 3 \cot ^2\left( \sqrt{\delta } \left( -\frac{\eta _2^2 \rho _2 t}{\eta _1}+\eta _1 \left( x-t \left( 4 \delta \eta _1^2+\rho _1\right) \right) +\eta _2 y\right) \right) +1\right) , \end{aligned}$$
  19 $$\begin{aligned} \mathcal {B}_{\text {III}}(x,y,t)= & 2 \delta \eta _1^2 \left( \frac{3}{\sin \left( \sqrt{\delta } \left( -\frac{\eta _2^2 \rho _2 t}{\eta _1}+\eta _1 \left( x-t \left( \delta \eta _1^2+\rho _1\right) \right) +\eta _2 y\right) \right) -1}+1\right) , \end{aligned}$$
  20 $$\begin{aligned} \mathcal {B}_{\text {III}}(x,y,t)= & \delta \eta _1^2 \left( 2-3\sec ^2\left( \frac{1}{2} \sqrt{\delta } \left( -\frac{\eta _2^2 \rho _2 t}{\eta _1}+\eta _1 \left( x-t \left( \delta \eta _1^2+\rho _1\right) \right) +\eta _2 y\right) \right) \right) , \end{aligned}$$
  21 $$\begin{aligned} \mathcal {B}_{\text {IV}}(x,y,t)= & \frac{6 \delta \eta _1^2}{\sin \left( \sqrt{\delta } \left( \delta \eta _1^3 t-\frac{\eta _2^2 \rho _2 t}{\eta _1}+\eta _1 \left( x-\rho _1 t\right) +\eta _2 y\right) \right) -1}, \end{aligned}$$
  22 $$\begin{aligned} \mathcal {B}_{\text {IV}}(x,y,t)= & -3 \delta \eta _1^2 \sec ^2\left( \frac{1}{2} \sqrt{\delta } \left( \delta \eta _1^3 t-\frac{\eta _2^2 \rho _2 t}{\eta _1}+\eta _1 \left( x-\rho _1 t\right) +\eta _2 y\right) \right) . \end{aligned}$$
3. **Soliton wave via the GRat method:**
  Evaluating the value of the above–mentioned parameters through the UF method, gets
  **Set I**
  $$\begin{aligned} a_1\rightarrow 0,c\rightarrow -\frac{1}{3} \eta _1 \left( a_0+3 \rho _1\right) -\frac{\eta _2^2 \rho _2}{\eta _1},\zeta \rightarrow \frac{i a_0}{2 \sqrt{3} \sqrt{a_2} \eta _1},\varrho \rightarrow \frac{i \sqrt{a_2}}{2 \sqrt{3} \eta _1},\, \text {where}\, (a_2<0). \end{aligned}$$
  **Set II**
  $$\begin{aligned} a_1\rightarrow 0,c\rightarrow \eta _1 \left( a_0-\rho _1\right) -\frac{\eta _2^2 \rho _2}{\eta _1},\zeta \rightarrow \frac{i \sqrt{3} a_0}{2 \sqrt{a_2} \eta _1},\varrho \rightarrow \frac{i \sqrt{a_2}}{2 \sqrt{3} \eta _1},\, \text {where}\, (a_2<0). \end{aligned}$$
  Thus, the solitary wave solutions of the investigated model are constructed for $\zeta \,\varrho>0$ by
  23 $$\begin{aligned} \mathcal {B}_{\text {I}}(x,y,t)= & \frac{a_2 \zeta }{\varrho } \tan ^2\left( \sqrt{\zeta \varrho } \left( t \left( -\frac{1}{3} \eta _1 \left( a_0+3 \rho _1\right) -\frac{\eta _2^2 \rho _2}{\eta _1}\right) +\eta _1 x+\eta _2 y+\Omega \right) \right) +a_0, \end{aligned}$$
  24 $$\begin{aligned} \mathcal {B}_{\text {I}}(x,y,t)= & \frac{a_2 \zeta }{\varrho } \cot ^2\left( \sqrt{\zeta \varrho } \left( t \left( -\frac{1}{3} \eta _1 \left( a_0+3 \rho _1\right) -\frac{\eta _2^2 \rho _2}{\eta _1}\right) +\eta _1 x+\eta _2 y+\Omega \right) \right) +a_0, \end{aligned}$$
  25 $$\begin{aligned} \mathcal {B}_{\text {II}}(x,y,t)= & \frac{a_2 \zeta }{\varrho } \tan ^2\left( \sqrt{\zeta \varrho } \left( t \left( \eta _1 \left( a_0-\rho _1\right) -\frac{\eta _2^2 \rho _2}{\eta _1}\right) +\eta _1 x+\eta _2 y+\Omega \right) \right) +a_0, \end{aligned}$$
  26 $$\begin{aligned} \mathcal {B}_{\text {II}}(x,y,t)= & \frac{a_2 \zeta }{\varrho } \cot ^2\left( \sqrt{\zeta \varrho } \left( t \left( \eta _1 \left( a_0-\rho _1\right) -\frac{\eta _2^2 \rho _2}{\eta _1}\right) +\eta _1 x+\eta _2 y+\Omega \right) \right) +a_0. \end{aligned}$$
  While, for $\zeta \,\varrho <0$, the solitary wave solutions of the investigated model are constructed by
  27 $$\begin{aligned} \mathcal {B}_{\text {I}}(x,y,t)= & \frac{a_2 \zeta }{\varrho } \tan ^2\left( \sqrt{\zeta } \sqrt{\varrho } \left( t \left( -\frac{1}{3} \eta _1 \left( a_0+3 \rho _1\right) -\frac{\eta _2^2 \rho _2}{\eta _1}\right) +\eta _1 x+\eta _2 y+\Omega \right) \right) +a_0, \end{aligned}$$
  28 $$\begin{aligned} \mathcal {B}_{\text {I}}(x,y,t)= & \frac{a_2 \zeta }{\varrho } \cot ^2\left( \sqrt{\zeta } \sqrt{\varrho } \left( t \left( -\frac{1}{3} \eta _1 \left( a_0+3 \rho _1\right) -\frac{\eta _2^2 \rho _2}{\eta _1}\right) +\eta _1 x+\eta _2 y+\Omega \right) \right) +a_0, \end{aligned}$$
  29 $$\begin{aligned} \mathcal {B}_{\text {II}}(x,y,t)= & \frac{a_2 \zeta }{\varrho }\, \tan ^2\left( \sqrt{\zeta } \sqrt{\varrho } \left( t \left( \eta _1 \left( a_0-\rho _1\right) -\frac{\eta _2^2 \rho _2}{\eta _1}\right) +\eta _1 x+\eta _2 y+\Omega \right) \right) +a_0, \end{aligned}$$
  30 $$\begin{aligned} \mathcal {B}_{\text {II}}(x,y,t)= & \frac{a_2 \zeta }{\varrho }\, \cot ^2\left( \sqrt{\zeta } \sqrt{\varrho } \left( t \left( \eta _1 \left( a_0-\rho _1\right) -\frac{\eta _2^2 \rho _2}{\eta _1}\right) +\eta _1 x+\eta _2 y+\Omega \right) \right) +a_0. \end{aligned}$$

### Solution’s accuracy

In this section, the accuracy of the previously constructed solutions is assessed using the HVI scheme. This approach involves comparing the analytical and numerical solutions of the (2+1) dimensional GS model to determine the absolute error over an extensive range, thereby ensuring the robustness of the solutions across a wide domain. Specifically, the accuracy of both solutions, given by Eqs. (11) and (15), (27), is examined, as detailed in Tables 2, 3, and 4.

31 $$\begin{aligned} & \begin{aligned} u_{\text {Approx. Sol}} \Bigg |_{\text {Eq.} (11)}=&\frac{1}{1073741824} \Bigg ( 9\, \text {sech}^2(\frac{1}{8} \sqrt{3} (y-x)) (9 t (6561 t^2 \text {sech}^8(\frac{1}{8} \sqrt{3} (y-x))-81 t\\ &\times (135 t+1792) \text {sech}^6(\frac{1}{8} \sqrt{3} (y-x))+5184 t (t+44) \text {sech}^4(\frac{1}{8} \sqrt{3} (y-x))-24\\&\times \text {sech}^2(\frac{1}{8} \sqrt{3} (y-x)) (9 t (3 t+416)+8192 \sqrt{3} \tanh (\frac{1}{8} \sqrt{3} (y-x))\\&-32768)+65536 \sqrt{3} \tanh (\frac{1}{8} \sqrt{3} (y-x)))+512 (9 t (9 t-1024)+131072)) \Bigg ). \end{aligned} \end{aligned}$$

32 $$\begin{aligned} & \begin{aligned} u_{\text {Approx. Sol}} \Bigg |_{\text {Eq.} (15)}=12&\text {sech}^2(x+2 y) \Bigg (24 t (\text {sech}(x+2 y) (3 \text {sech}(x+2 y) (2 \text {sech}(x+2 y) (t \text {sech}(x+2 y)\\&(1296 t \text {sech}^4(x+2 y)-6 (360 t+7) \text {sech}^2(x+2 y)+1024 t+71)+\text {sech}(x)\\&\times \sinh (2 y))-2 t (128 t+31)+2 \tanh (x)+1)-2 \text {sech}(x) \sinh (2 y))\\&-2 \tanh (x))+48 t (6 t-1)+1\Bigg )-8. \end{aligned} \end{aligned}$$

33 $$\begin{aligned} & \begin{aligned} u_{\text {Approx. Sol}} \Bigg |_{\text {Eq.} (27)}=&\frac{1}{2916} \Bigg ( 5 \text {sech}^2(\frac{1}{2} \sqrt{\frac{5}{3}} (x-2 y)) (175 t (\text {sech}^2(\frac{1}{2} \sqrt{\frac{5}{3}} (x-2 y)) (175 t \text {sech}^2(\frac{1}{2} \sqrt{\frac{5}{3}} (x-2 y))\\&\times (27 \text {sech}^2(\frac{1}{2} \sqrt{\frac{5}{3}} (x-2 y)) (75 t \text {sech}^2(\frac{1}{2} \sqrt{\frac{5}{3}} (x-2 y))-125 t-3)+1600 t+243)\\&-25 t (1400 t+1161)+324 \sqrt{15} \tanh (\frac{1}{2} \sqrt{\frac{5}{3}} (x-2 y))+972)-108\\&\times \sqrt{15} \tanh (\frac{1}{2} \sqrt{\frac{5}{3}} (x-2 y)))+3150 t (175 t-36)+2916)\Bigg ). \end{aligned} \end{aligned}$$

**Table 2:** Accuracy of Eq. (11) solutions via Khat III and HVI: comparison of analytical, numerical results, and absolute errors for various $x$.

**Value of**$x$	**Analy. Sol.**	**Num. Sol.**	**|Error|**	**Value of**$x$	**Analy. Sol.**	**Num. Sol.**	**|Error|**
0	0.536935422650468	0.536935422672529	2.20611540364244E-11	0.515625	0.556358828685349	0.556358828697608	1.22586222981402E-11
0.015625	0.537704567891936	0.537704567913726	2.17896695352771E-11	0.53125	0.556745978872475	0.556745978884413	1.19386983648742E-11
0.03125	0.538463033633493	0.538463033655009	2.15163733054409E-11	0.546875	0.557120777300937	0.557120777312555	1.16177205826216E-11
0.046875	0.539210754957689	0.539210754978930	2.12412838839112E-11	0.5625	0.557483190166818	0.557483190178114	1.12957159705921E-11
0.0625	0.539947667729222	0.539947667750187	2.09644201139946E-11	0.578125	0.557833184750669	0.557833184761642	1.09727117383391E-11
0.078125	0.540673708606342	0.540673708627028	2.06858011430802E-11	0.59375	0.558170729423755	0.558170729434404	1.06487352810346E-11
0.09375	0.541388815052143	0.541388815072548	2.04054464203240E-11	0.609375	0.558495793654102	0.558495793664426	1.03238141746855E-11
0.109375	0.542092925345749	0.542092925365873	2.01233756942489E-11	0.625	0.558808348012338	0.558808348022336	9.99797617129256E-12
0.125	0.542785978593386	0.542785978613226	1.98396090102579E-11	0.640625	0.559108364177334	0.559108364187005	9.67124919395428E-12
0.140625	0.543467914739330	0.543467914758884	1.95541667080634E-11	0.65625	0.559395814941639	0.559395814950982	9.34366133191621E-12
0.15625	0.544138674576741	0.544138674596008	1.92670694190316E-11	0.671875	0.559670674216698	0.559670674225713	9.01524083556790E-12
0.171875	0.544798199758372	0.544798199777350	1.89783380634419E-11	0.6875	0.559932917037878	0.559932917046564	8.68601611138853E-12
0.1875	0.545446432807145	0.545446432825833	1.86879938476640E-11	0.703125	0.560182519569262	0.560182519577618	8.35601571684289E-12
0.203125	0.546083317126606	0.546083317145002	1.83960582612503E-11	0.71875	0.560419459108251	0.560419459116276	8.02526835522917E-12
0.21875	0.546708797011233	0.546708797029336	1.81025530739465E-11	0.734375	0.560643714089933	0.560643714097627	7.69380287048021E-12
0.234375	0.547322817656625	0.547322817674432	1.78075003326197E-11	0.75	0.560855264091250	0.560855264098612	7.36164824191975E-12
0.25	0.547925325169531	0.547925325187042	1.75109223581052E-11	0.765625	0.561054089834942	0.561054089841971	7.02883357897519E-12
0.265625	0.548516266577751	0.548516266594964	1.72128417419717E-11	0.78125	0.561240173193274	0.561240173199969	6.69538811584863E-12
0.28125	0.549095589839884	0.549095589856798	1.69132813432074E-11	0.796875	0.561413497191539	0.561413497197900	6.36134120614775E-12
0.296875	0.549663243854930	0.549663243871542	1.66122642848252E-11	0.8125	0.561574046011351	0.561574046017378	6.02672231747824E-12
0.3125	0.550219178471731	0.550219178488041	1.63098139503906E-11	0.828125	0.561721804993704	0.561721804999396	5.69156102599931E-12
0.328125	0.550763344498273	0.550763344514279	1.60059539804701E-11	0.84375	0.561856760641814	0.561856760647170	5.35588701094425E-12
0.34375	0.551295693710812	0.551295693726513	1.57007082690034E-11	0.859375	0.561978900623740	0.561978900628759	5.01973004910750E-12
0.359375	0.551816178862853	0.551816178878247	1.53941009595993E-11	0.875	0.562088213774771	0.562088213779454	4.68312000930008E-12
0.375	0.552324753693957	0.552324753709043	1.50861564417550E-11	0.890625	0.562184690099601	0.562184690103948	4.34608684677513E-12
0.390625	0.552821372938384	0.552821372953161	1.47768993470025E-11	0.90625	0.562268320774265	0.562268320778273	4.00866059762529E-12
0.40625	0.553305992333566	0.553305992348033	1.44663545449798E-11	0.921875	0.562339098147852	0.562339098151523	3.67087137315376E-12
0.421875	0.553778568628412	0.553778568642566	1.41545471394308E-11	0.9375	0.562397015743999	0.562397015747331	3.33274935422067E-12
0.4375	0.554239059591427	0.554239059605269	1.38415024641326E-11	0.953125	0.562442068262141	0.562442068265135	2.99432478556678E-12
0.453125	0.554687424018672	0.554687424032200	1.35272460787527E-11	0.96875	0.562474251578550	0.562474251581206	2.65562797011618E-12
0.46875	0.555123621741525	0.555123621754737	1.32118037646369E-11	0.984375	0.562493562747132	0.562493562749448	2.31668926325983E-12
0.484375	0.555547613634271	0.555547613647166	1.28952015205284E-11	1	0.5625	0.5625	1.97753906712179E-12

**Table 3:** Accuracy of Eq. (15) solutions via Khat II and HVI: comparison of analytical, numerical results, and absolute errors for various $x$.

**Value of**$x$	**Analy. Sol.**	**Num. Sol.**	**|Error|**	**Value of**$x$	**Analy. Sol.**	**Num. Sol.**	**|Error|**
0	−7.15219010078125	−7.15219010849183	7.7105809141665E-09	0.515625	−7.69058374582722	−7.69058374897399	3.1467662992860E-09
0.015625	−7.17736426485051	−7.17736427237301	7.5224962303405E-09	0.53125	−7.69998371478047	−7.69998371783734	3.0568721775707E-09
0.03125	−7.20181764823111	−7.20181765556856	7.3374474278174E-09	0.546875	−7.70910166711680	−7.70910167008613	2.9693357998468E-09
0.046875	−7.22556929316021	−7.22556930031569	7.1554884730058E-09	0.5625	−7.71794584984695	−7.71794585273106	2.8841083472433E-09
0.0625	−7.24863783522985	−7.24863784220651	6.9766643583142E-09	0.578125	−7.72652428176185	−7.72652428456299	2.8011411530012E-09
0.078125	−7.27104150595628	−7.27104151275729	6.8010116893468E-09	0.59375	−7.73484475895443	−7.73484476167482	2.7203857720028E-09
0.09375	−7.29279813579458	−7.29279814242314	6.6285592456138E-09	0.609375	−7.74291486025838	−7.74291486290018	2.6417940443156E-09
0.109375	−7.31392515755974	−7.31392516401907	6.4593285152964E-09	0.625	−7.75074195260152	−7.75074195516684	2.5653181531154E-09
0.125	−7.33443961021736	−7.33443961651070	6.2933342046657E-09	0.640625	−7.75833319627170	−7.75833319876261	2.4909106773294E-09
0.140625	−7.35435814300938	−7.35435814913997	6.1305847227952E-09	0.65625	−7.76569555009355	−7.76569555251207	2.4185246393308E-09
0.15625	−7.37369701988214	−7.37369702585322	5.9710826422451E-09	0.671875	−7.77283577651477	−7.77283577886288	2.3481135479944E-09
0.171875	−7.39247212418609	−7.39247213000092	5.8148251364253E-09	0.6875	−7.77976044660068	−7.77976044888031	2.2796314374091E-09
0.1875	−7.41069896361841	−7.41069896928021	5.6618043943644E-09	0.703125	−7.78647594493623	−7.78647594714927	2.2130329015298E-09
0.203125	−7.42839267538131	−7.42839268089332	5.5120080136318E-09	0.71875	−7.79298847443487	−7.79298847658314	2.1482731250329E-09
0.21875	−7.44556803153088	−7.44556803689630	5.3654193721665E-09	0.734375	−7.79930406105366	−7.79930406313897	2.0853079106290E-09
0.234375	−7.46223944449255	−7.46223944971457	5.2220179797760E-09	0.75	−7.80542855841467	−7.80542856043876	2.0240937030710E-09
0.25	−7.47842097272105	−7.47842097780283	5.0817798100680E-09	0.765625	−7.81136765233227	−7.81136765429686	1.9645876100838E-09
0.265625	−7.49412632648417	−7.49412633142885	4.9446776135769E-09	0.78125	−7.81712686524675	−7.81712686715350	1.9067474204289E-09
0.28125	−7.50936887375093	−7.50936887856161	4.8106812128424E-09	0.796875	−7.82271156056428	−7.82271156241481	1.8505316193055E-09
0.296875	−7.52416164616613	−7.52416165084589	4.6797577801864E-09	0.8125	−7.82812694690370	−7.82812694869960	1.7958994012793E-09
0.3125	−7.53851734509460	−7.53851734964647	4.5518720989288E-09	0.828125	−7.83337808225080	−7.83337808399361	1.7428106809175E-09
0.328125	−7.55244834771953	−7.55244835214651	4.4269868087651E-09	0.84375	−7.83846987802045	−7.83846987971168	1.6912261013001E-09
0.34375	−7.56596671318042	−7.56596671748549	4.3050626360171E-09	0.859375	−7.84340710302759	−7.84340710466870	1.6411070405666E-09
0.359375	−7.57908418873743	−7.57908419292348	4.1860586094522E-09	0.875	−7.84819438736781	−7.84819438896023	1.5924156166488E-09
0.375	−7.59181221594948	−7.59181222001941	4.0699322623461E-09	0.890625	−7.85283622620847	−7.85283622775359	1.5451146903305E-09
0.390625	−7.60416193685508	−7.60416194081172	3.9566398214509E-09	0.90625	−7.85733698349134	−7.85733698499051	1.4991678667662E-09
0.40625	−7.61614420014505	−7.61614420399119	3.8461363835050E-09	0.921875	−7.86170089554786	−7.86170089700240	1.4545394955854E-09
0.421875	−7.62776956731765	−7.62776957105602	3.7383760799077E-09	0.9375	−7.86593207462822	−7.86593207603941	1.4111946696977E-09
0.4375	−7.63904831880721	−7.63904832244052	3.6333122301555E-09	0.953125	−7.87003451234529	−7.87003451371439	1.3690992229095E-09
0.453125	−7.64999046007825	−7.64999046360915	3.5308974846222E-09	0.96875	−7.87401208303483	−7.87401208436305	1.3282197264550E-09
0.46875	−7.66060572767751	−7.66060573110859	3.4310839572378E-09	0.984375	−7.87786854703312	−7.87786854832164	1.2885234845386E-09
0.484375	−7.67090359523734	−7.67090359857117	3.3338233486067E-09	1	−7.88160755387335	−7.88160755512333	1.2499785289771E-09

**Table 4:** Accuracy of Eq. (14) solutions via GRat and HVI: comparison of analytical, numerical results, and absolute errors for various $x$.

**Value of** $x$	**Analy. Sol.**	**Num. Sol.**	**|Error|**	**Value of** $x$	**Analy. Sol.**	**Num. Sol.**	**|Error|**
0	1.30727195183026	1.30727195104044	7.89818323140150E-10	0.515625	2.23637345031803	2.23637344853350	1.78453748179146E-09
0.015625	1.33009597781315	1.33009597699854	8.14607276997109E-10	0.53125	2.27006087399251	2.27006087217852	1.81399507638128E-09
0.03125	1.35324525137045	1.35324525053054	8.39904931522343E-10	0.546875	2.30404291104266	2.30404290919991	1.84274697126368E-09
0.046875	1.37672160882652	1.37672160796081	8.65706731536448E-10	0.5625	2.33831441396632	2.33831441209562	1.87070725247819E-09
0.0625	1.40052677882934	1.40052677793733	8.92006938746664E-10	0.578125	2.37286990589146	2.37286990399368	1.89778643124900E-09
0.078125	1.42466237683887	1.42466237592008	9.18798560890546E-10	0.59375	2.40770357425605	2.40770357233216	1.92389143733586E-09
0.09375	1.44912989948968	1.44912989854361	9.46073278808331E-10	0.609375	2.44280926467375	2.44280926272483	1.94892562274130E-09
0.109375	1.47393071882943	1.47393071785561	9.73821371504459E-10	0.625	2.47818047500467	2.47818047303188	1.97278877650587E-09
0.125	1.49906607643540	1.49906607543337	1.00203163927137E-09	0.640625	2.51381034965099	2.51381034765562	1.99537715133067E-09
0.140625	1.52453707741128	1.52453707638059	1.03069132496217E-09	0.65625	2.54969167409788	2.54969167208130	2.01658350277090E-09
0.15625	1.55034468426704	1.55034468320726	1.05978603351301E-09	0.671875	2.58581686972044	2.58581686768415	2.03629714174528E-09
0.171875	1.57648971068495	1.57648970959565	1.08929964983146E-09	0.6875	2.62217798887806	2.62217798682365	2.05440400110318E-09
0.1875	1.60297281517528	1.60297281405607	1.11921425518279E-09	0.703125	2.65876671031764	2.65876670824686	2.07078671698453E-09
0.203125	1.62979449462559	1.62979449347608	1.14951004222353E-09	0.71875	2.69557433490804	2.69557433282271	2.08532472569621E-09
0.21875	1.65695507774796	1.65695507656779	1.18016522884911E-09	0.734375	2.73259178172769	2.73259177962979	2.09789437681233E-09
0.234375	1.68445471842899	1.68445471721784	1.21115597104127E-09	0.75	2.76980958452842	2.76980958242005	2.10836906318497E-09
0.25	1.71229338898781	1.71229338774536	1.24245627492026E-09	0.765625	2.80721788859805	2.80721788648143	2.11661936852527E-09
0.265625	1.74047087334780	1.74047087207376	1.27403790822758E-09	0.78125	2.84480644804489	2.84480644592238	2.12251323318283E-09
0.28125	1.76898676012836	1.76898675882249	1.30587031148569E-09	0.796875	2.88256462352726	2.88256462140134	2.12591613871334E-09
0.296875	1.79784043566342	1.79784043432550	1.33792050910348E-09	0.8125	2.92048138045114	2.92048137832445	2.12669131178043E-09
0.3125	1.82703107695402	1.82703107558386	1.37015302071863E-09	0.828125	2.95854528765920	2.95854528553450	2.12469994788723E-09
0.328125	1.85655764456265	1.85655764316012	1.40252977309149E-09	0.84375	2.99674451663408	2.99674451451428	2.11980145537630E-09
0.34375	1.88641887545796	1.88641887402295	1.43501001288903E-09	0.859375	3.03506684123902	3.03506683912716	2.11185372007295E-09
0.359375	1.91661327581859	1.91661327435104	1.46755022072166E-09	0.875	3.07349963801815	3.07349963591743	2.10071339087648E-09
0.375	1.94713911380572	1.94713911230562	1.50010402682096E-09	0.890625	3.11202988707905	3.11202988499281	2.08623618652658E-09
0.390625	1.97799441231455	1.97799441078193	1.53262212877129E-09	0.90625	3.15064417357921	3.15064417151093	2.06827722368783E-09
0.40625	2.00917694171525	2.00917694015019	1.56505221173401E-09	0.921875	3.18932868983782	3.18932868779113	2.04669136640388E-09
0.421875	2.04068421259490	2.04068421099756	1.59733887162851E-09	0.9375	3.22806923809364	3.22806923607231	2.02133359687484E-09
0.4375	2.07251346851236	2.07251346688294	1.62942354176000E-09	0.953125	3.26685123392899	3.26685123193693	1.99205940740639E-09
0.453125	2.10466167877848	2.10466167711723	1.66124442340949E-09	0.96875	3.30565971037914	3.30565970842042	1.95872521326761E-09
0.46875	2.13712553127512	2.13712552958239	1.69273642092637E-09	0.984375	3.34447932274543	3.34447932082424	1.92118878607644E-09
0.484375	2.16990142532668	2.16990142360285	1.72383108188923E-09	1	3.38329435412947	3.38329435225016	1.87930970720793E-09

## Solutions’ graphical representation and calculated tables’ interpretation

This section provides a detailed analysis of the graphical and tabular data, elucidating the physical significance and implications of the investigated models. The figures and tables are crucial for comprehending the characteristics and behaviors of the wave functions described by the equations in the study.

**Fig. 5:**
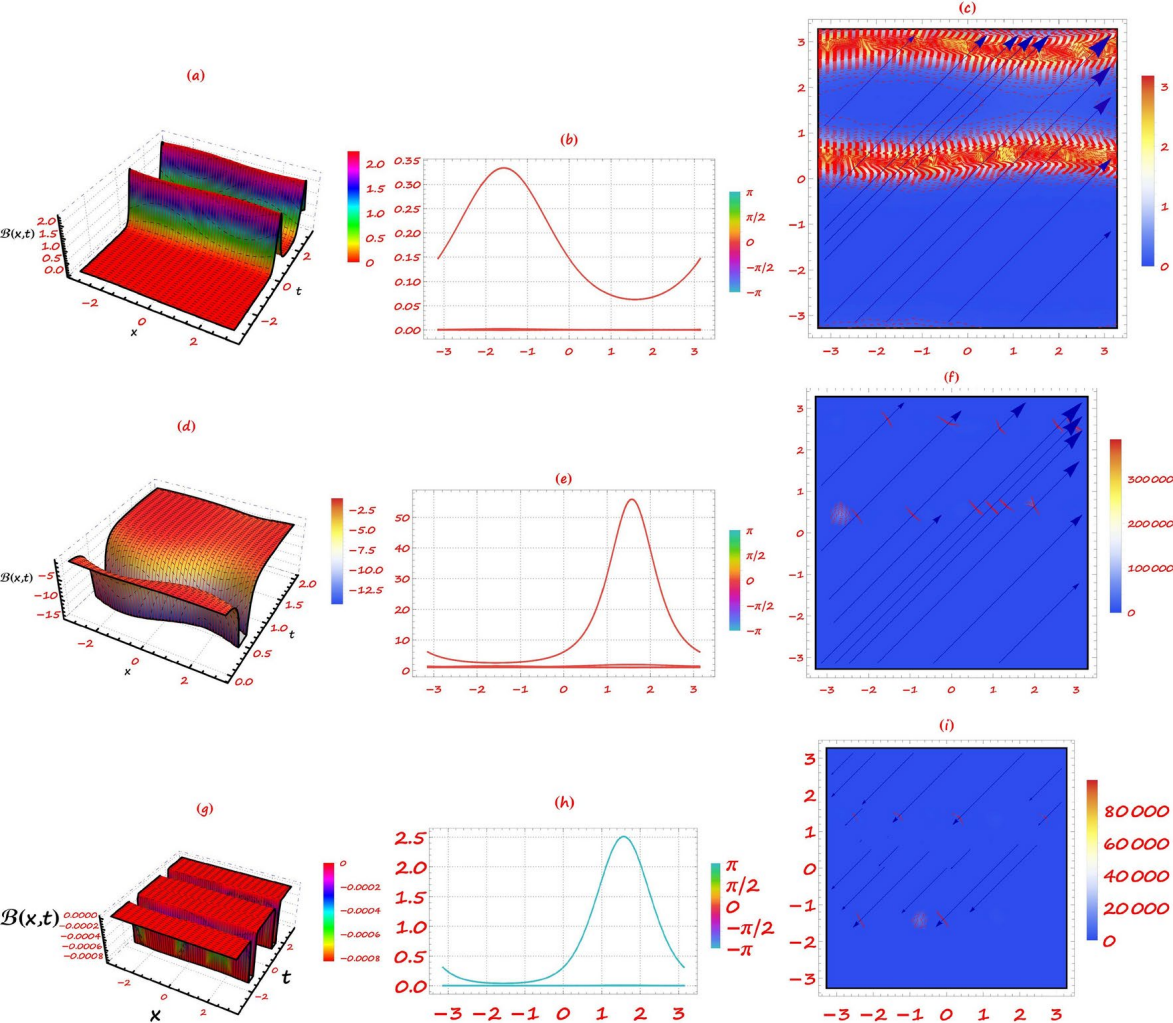
3D (**a**, **d**, **g**), 2D (**b**, **e**, **h**), and density (**c**, **f**, **i**) plots show periodic wave features from Eqs. (11) (a-c), (13) (d-f), and (14) (g-i). 3D plots reveal spatial structure and amplitude, 2D highlights periodicity, and density uses color (red: high, blue: low) to show intensity. Arrows indicate key points.

**Fig. 6:**
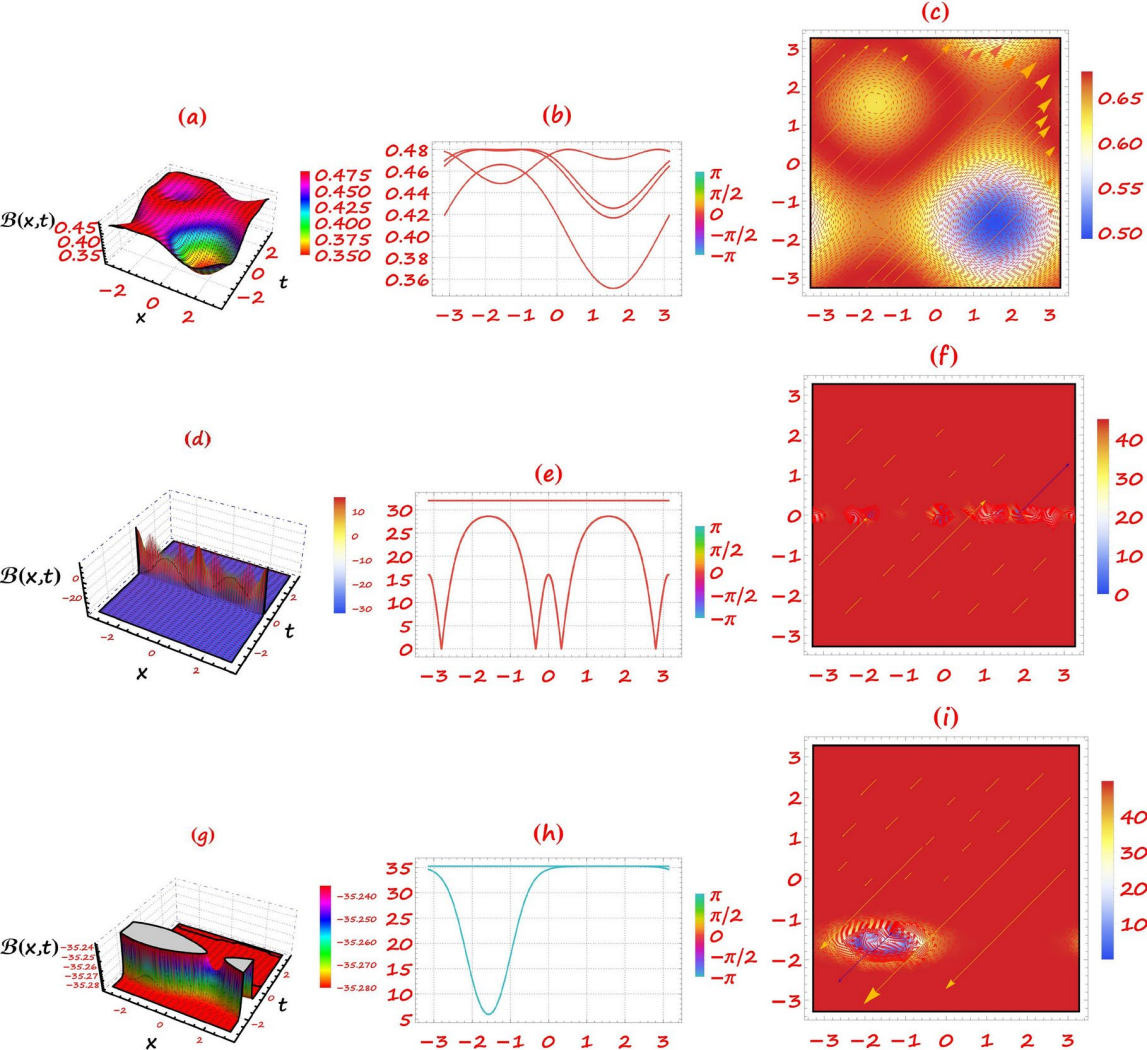
3D (a,d), 2D (b,e), and density (c,f) plots illustrate periodic wave features from Eqs. (15) (**a**-**c**) and (17) (**d**-**f**). 3D shows spatial structure, 2D offers an overhead view, and density uses color (red: high, blue: low) for intensity. Arrows mark key periodicity and phase points.

**Fig. 7:**
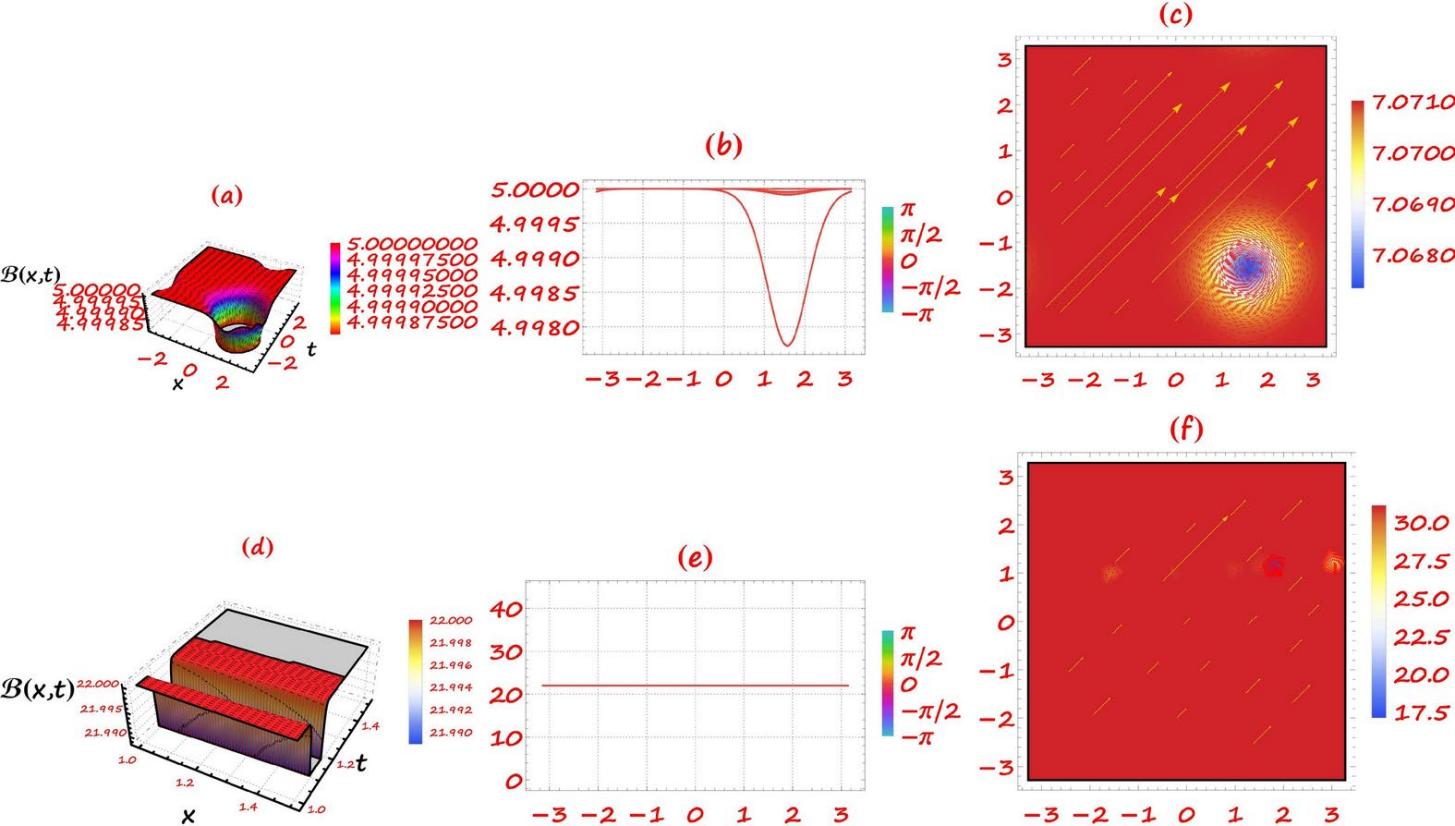
3D (a,d), 2D (b,e), and density (c,f) plots illustrate periodic wave features from Eqs. (27) (**a**-**c**) and (29) (**d**-**f**). 3D shows spatial structure, 2D highlights periodicity, and density uses color (red: high, blue: low) for intensity. Arrows mark phase transitions and amplitude changes.

**Fig. 8:**
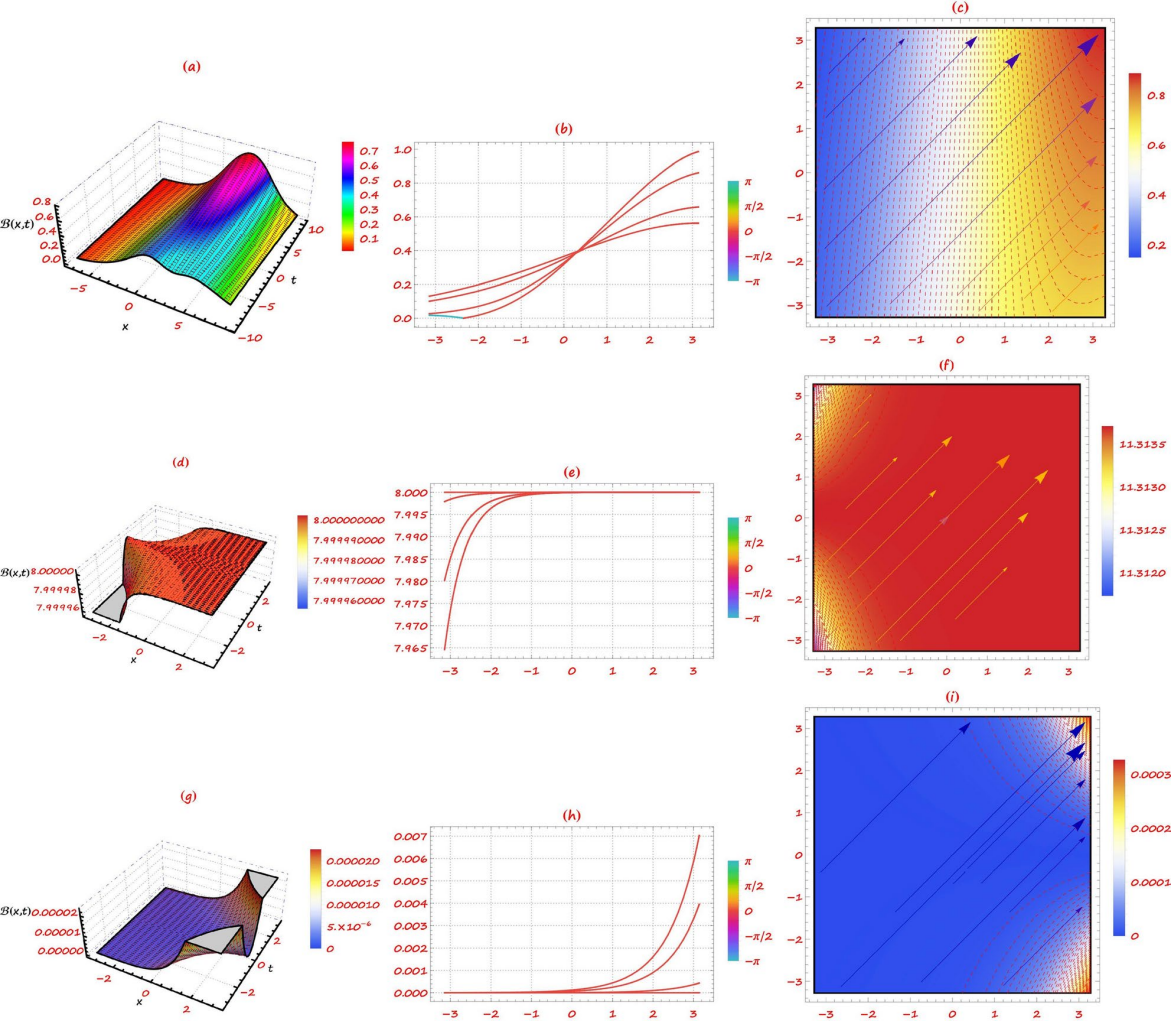
3D (a,d,g), 2D (b,e,h), and density (c,f,i) plots illustrate periodic wave features from Eqs. (31) (**a**-**c**), (32) (**d**-**f**), and (33) (g-i). 3D shows wave structure, 2D offers a top-down view, and density uses color (red: high, blue: low) for intensity. Arrows mark key phase and amplitude changes.

**Fig. 9:**
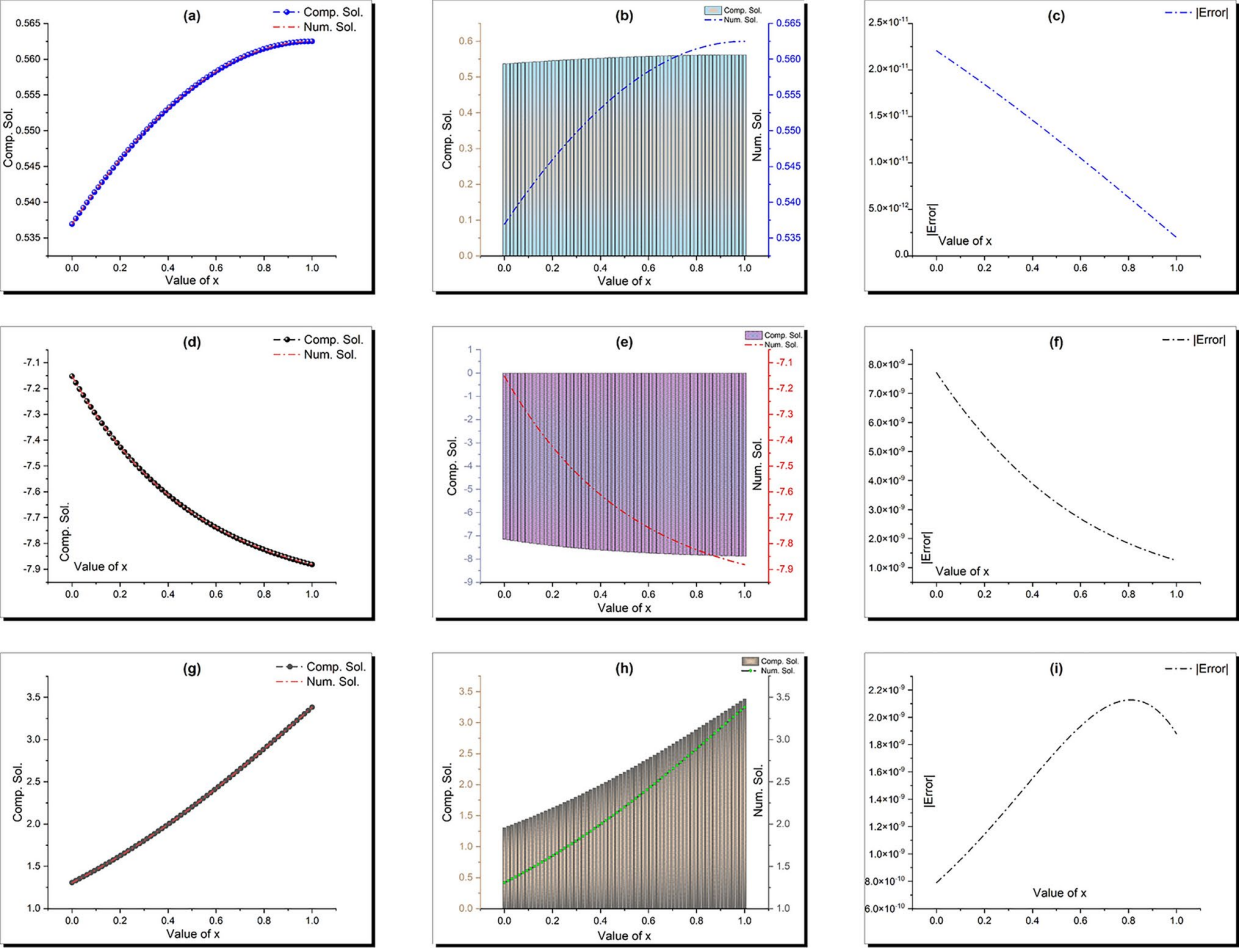
Comparative graphs show alignment between analytical and numerical solutions. Sub-figures (**a**-**c**) compare the Khat III method (Eq. (11)) with HVI scheme solutions. Sub-figures (**d**-**f**) compare the Khat II method (Eq. (15)) with HVI solutions. Sub-figures (**g**-**i**) compare the GRat method (Eq. (27)) with Adomian decomposition solutions. Colors indicate error magnitudes, with arrows marking key agreements or deviations.

**Fig. 10:**
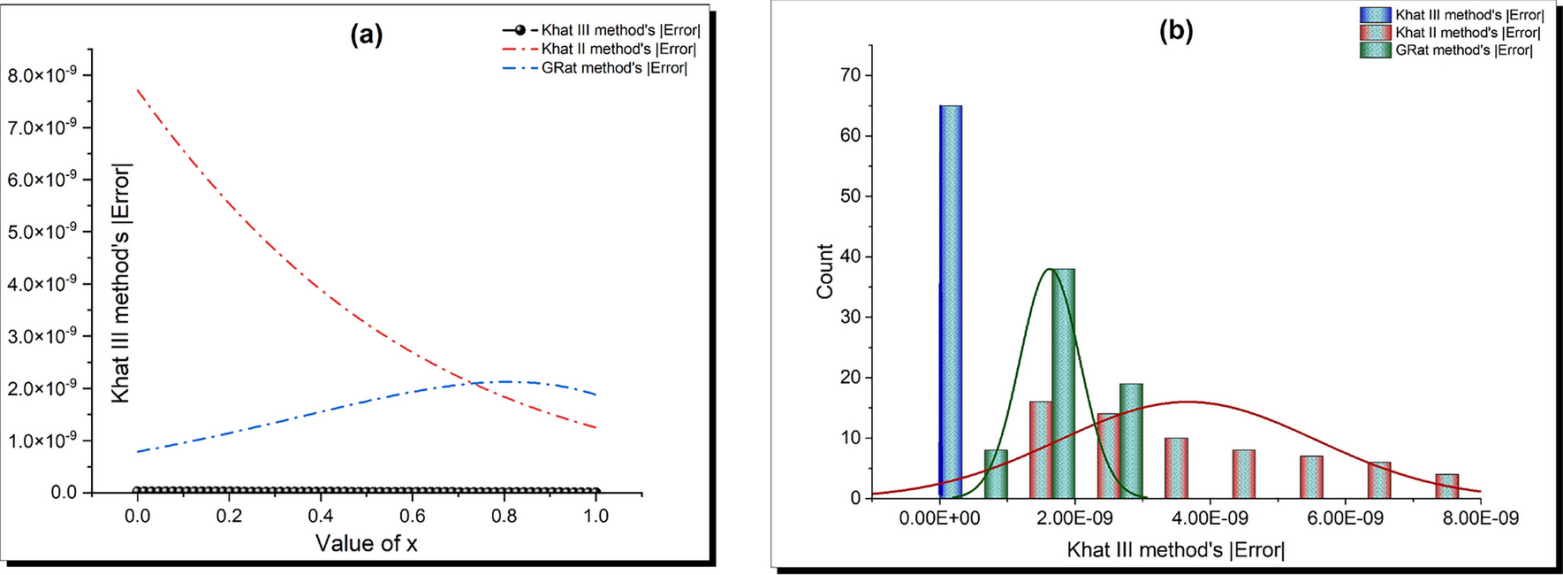
Comparison of analytical methods highlights the superiority of the Khat III method in accurately modeling wave phenomena.

**Fig. 11:**
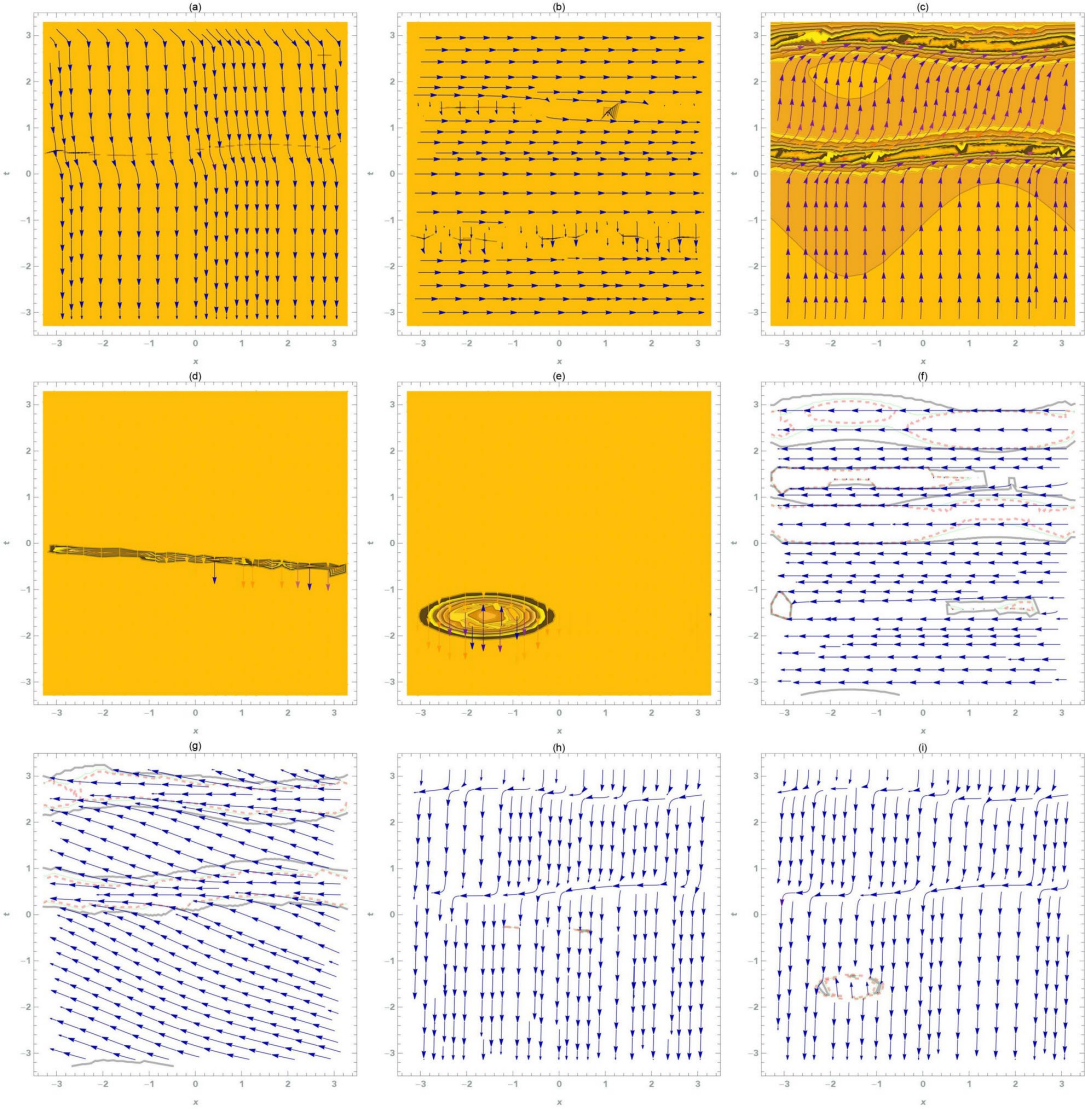
Stream plot of the solitary wave solution to the (2+1)-dimensional GS Model, showing the spatiotemporal evolution of wave structures in graphene. Streamline density and orientation reflect wave amplitudes and phase characteristics, revealing nonlinear dynamics and complex wave patterns in graphene sheets.

**Fig. 12:**
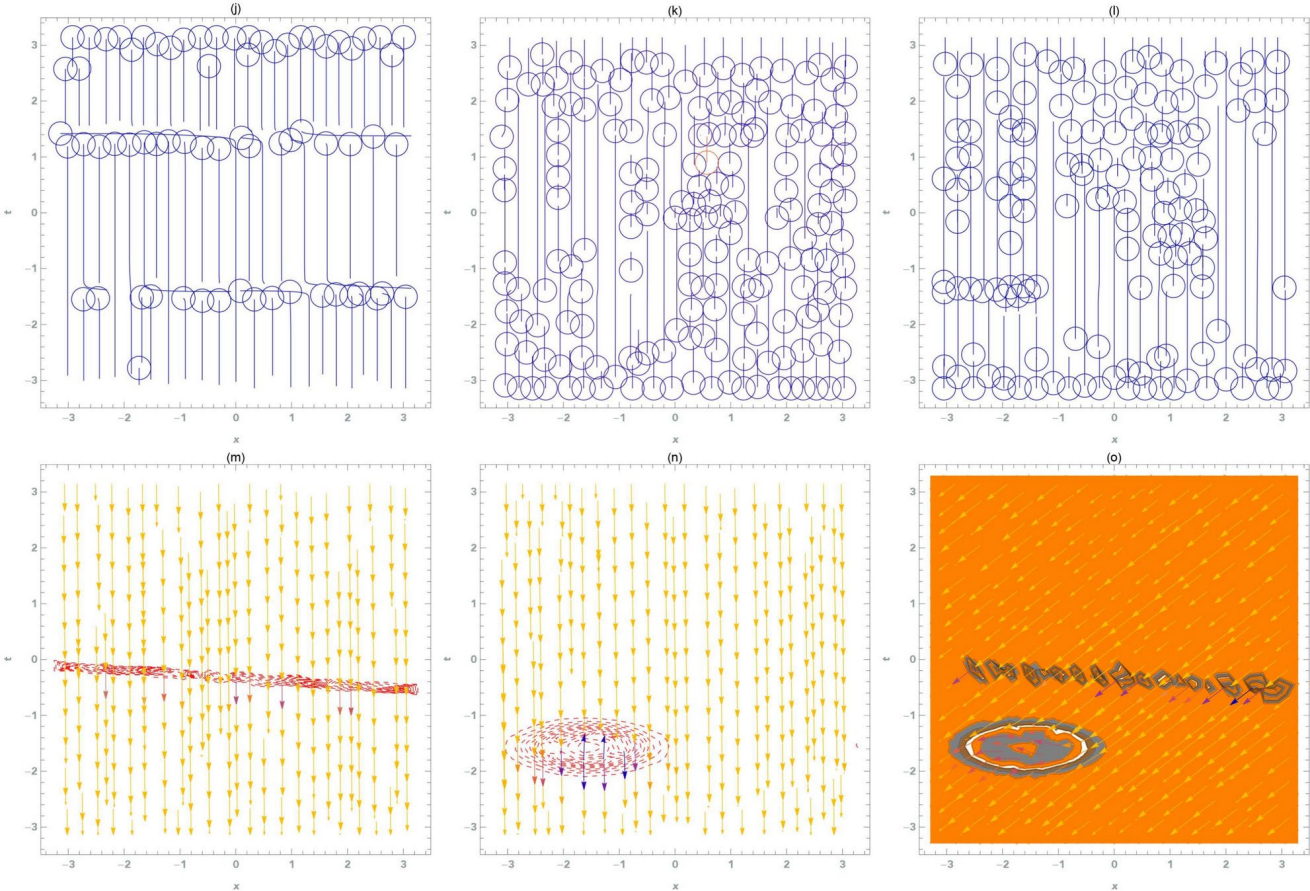
Stream plot of the solitary wave solution to the (2+1)-Dimensional GS Model, showing the spatiotemporal evolution of wave structures in graphene. Streamline density and orientation represent wave amplitudes and phases, aiding in the analysis of interactions, nonlinear dynamics, and complex patterns in graphene sheets.

- $$\bullet$$ Represented graphs:
  - 1. Fig. 1: This figure presents a series of bifurcation diagrams illustrating the phase portraits of the planar dynamical system described by Eq. (5). Each diagram corresponds to different parameter values, as outlined in Table 1. The equilibrium points, denoted as $\mathcal {E}_{1}$ and $\mathcal {E}_{2}$, are classified as either centers or saddles based on the determinant and trace of the Jacobian matrix.
    **Diagrams (a) to (j)**: These diagrams show the phase orbits for various combinations of $\eta _1$, $\eta _2$, $\rho _1$, $\rho _2$, and $c$. The equilibrium points are marked, and the orbits illustrate the system’s behavior around these points. For instance, in diagram (a), $\mathcal {E}_{1}$ is a center, indicating stable oscillations, while $\mathcal {E}_{2}$ is a saddle, indicating instability in certain directions.
  - 2. Fig. 2: This figure displays the phase portraits of the perturbed nonlinear dynamical system described by Eq. (10). The perturbation term $f_{0} \,cos (\mu \,\mathfrak {Z})$ introduces chaotic and quasi-periodic behaviors, which are visually represented here.
    **Diagrams (a) to (j)**: These diagrams illustrate the system’s behavior under varying amplitudes $f_{0}$ and frequencies $\mu$. The phase portraits show the system’s trajectories in state space, highlighting regions of stability and chaos. For example, diagram (a) might show a stable limit cycle, while diagram (b) could exhibit chaotic trajectories, indicating the system’s sensitivity to perturbations.
  - 3. Fig. 3: This figure presents the sensitivity analysis of the planar dynamical system described by Eq. (5). The sensitivity is assessed by examining the system’s response to small changes in initial conditions and parameter values.
    **Diagrams (a) to (i)**: These diagrams show the wave-forms $\psi (\mathfrak {Z})$ and $\upsilon (\mathfrak {Z})$ under different initial conditions. The visualizations demonstrate how slight variations in initial values significantly alter the system’s dynamics. For instance, diagram (a) might show a smooth waveform, while diagram (b) could exhibit erratic behavior, emphasizing the system’s sensitivity to initial conditions.
  - 4. Fig. 4: This figure further explores the sensitivity analysis by comparing waveforms from different initial positions. The diagrams highlight the complex interplay between parameters and system responses.
  - 5. **Diagrams (a) to (c)**: These diagrams compare the waveforms $\psi (\mathfrak {Z})$ and $\upsilon (\mathfrak {Z})$ from various initial positions. The findings illustrate the profound impact of small changes in initial values on the system’s dynamics, underscoring the importance of precise parameter selection in predicting system behavior.
  - 6. Fig. 5 :
    − Description: This figure comprises three-dimensional, two-dimensional, and density plots illustrating the periodic wave characteristics described by Eqs. (11), (13), (14).
    − Interpretation: The three-dimensional plot presents a spatial representation of the wave, where the height signifies amplitude, offering a clear visualization of the wave’s structure. The two-dimensional plot provides a top-down view, emphasizing the wave’s periodic nature. The density plot employs color gradients to depict variations in wave intensity, with red indicating high intensity and blue denoting low intensity. Arrows mark significant points of periodicity and phase shifts.
    − Physical Meaning: This figure aids in visualizing the spatial and intensity characteristics of a periodic wave function, enhancing understanding of its periodic behavior and amplitude variations.
  - 7. Fig. 6 :
    − Description: Similar to Fig. 5, this figure depicts the periodic wave characteristics described by Eqs. (15), (17) through three-dimensional, two-dimensional, and density plots.
    − Interpretation: The three-dimensional plot displays the spatial structure of the wave, with height representing amplitude. The two-dimensional plot offers an overhead perspective of the wave’s periodic features. The density plot highlights intensity variations using a color gradient from red (high intensity) to blue (low intensity). Arrows indicate key points of periodicity and phase changes.
    − Physical Meaning: This figure aids in understanding the spatial configuration and intensity variations of another periodic wave function, facilitating comprehension of its dynamic properties.
  - 8. Fig. 7 :
    − Description: This figure illustrates the periodic wave characteristics described by Eq. (27), (29) using three-dimensional, two-dimensional, and density plots.
    − Interpretation: The three-dimensional plot depicts the wave’s spatial configuration, with amplitude represented by height. The two-dimensional plot provides a top-down view, highlighting periodic features. The density plot uses color gradients to indicate intensity variations, with red denoting higher intensities and blue indicating lower ones. Arrows mark critical regions of phase transition and amplitude modulation.
    − Physical Meaning: This figure provides a comprehensive view of a periodic wave’s spatial structure and intensity variations, aiding in the analysis of its properties.
  - 9. Fig. 8 :
    − Description: This figure presents the periodic wave characteristics described by Eqs. (31), (32), and (33) through three-dimensional, two-dimensional, and density plots.
    − Interpretation: The three-dimensional plot visualizes the complex structure of the wave. The two-dimensional plot offers a top-down view, and the density plot uses color coding from red (high intensity) to blue (low intensity) to highlight intensity variations. Arrows denote significant areas of phase and amplitude changes.
    − Physical Meaning: This figure explores the complex behavior of a periodic wave, providing insights into different aspects of the wave’s properties through various visual representations.
  - 10. Fig. 9 :
    − Description: Comparative graphs demonstrating the alignment between analytical and numerical solutions for Eqs. (11), (15), and (29).
    − Interpretation: Sub-figures (a, b, c) compare the analytical solution via the KhatIII method (Eq. (11)) with numerical solutions obtained using the HVI scheme. Sub-figures (d, e, f) compare the analytical solution from the KhatII method (Eq. (15)) with numerical solutions from the Adomian decomposition scheme. Sub-figures (g, h, i) compare the analytical solution from the GRat method (Eq. (27)) with numerical solutions from the Adomian decomposition scheme. Colors in the graphs indicate error magnitudes, and arrows highlight areas of significant agreement or deviation.
    − Physical Meaning: This figure validates the analytical methods by comparing them with numerical solutions, highlighting areas of agreement and discrepancies, thereby assessing the accuracy of the analytical approaches.
  - 11. Fig. 10 :
    − Description: Graphical representation illustrating the superiority of the KhatIII method over other analytical schemes.
    − Interpretation: The figure compares the performance of different analytical methods, demonstrating the effectiveness of the KhatIII method in accurately modeling wave phenomena.
    − Physical Meaning: This figure illustrates the comparative advantages of the KhatIII method, reinforcing its reliability in wave modeling.
  - 12. Figs. 11, and (12) :
    − Description: Stream plot of the solitary wave solution to the (2+1)-dimensional GS Model.
    − Interpretation: The streamlines represent the propagation of solitary waves, with their density and orientation indicating wave amplitudes and phase characteristics.
    − Physical Meaning: This figure visualizes the solitary wave dynamics in a graphene sheet, providing insights into how these waves influence the material’s electronic and structural properties.
- $$\bullet$$ Calculated tables:
  - 1. Table 2 :
    − Description: Comparison of Analytical and Numerical Solutions for Eq. (11) Using Khat III and HVI Schemes.
    − Interpretation: This table lists various $x$ values, the corresponding analytical solutions, numerical solutions, and the absolute errors between these solutions.
    − Physical Meaning: The data in this table illustrate the accuracy and reliability of the Khat III and HVI schemes in solving Eq. (11), providing a quantitative assessment of the methods’ precision.
  - 2. Table 3 :
    − Description: Accuracy Assessment of Analytical and Numerical Solutions for Eq. (15) Using Khat II and HVI Schemes.
    − Interpretation: The table includes $x$ values, associated analytical solutions, numerical solutions, and the absolute errors.
    − Physical Meaning: This table demonstrates the precision of the KhatII and HVI schemes in solving Eq. (15), highlighting the robustness of these methods across different $x$ values.
  - 3. Table 4 :
    − Description: Evaluation of Solution Accuracy for Eq. (27) Using GRat and HVI Schemes.
    − Interpretation: The table documents various $x$ values alongside their respective analytical solutions, numerical solutions, and the absolute errors between them.
    − Physical Meaning: This evaluation indicates the effectiveness of the GRat and HVI methods in producing precise solutions for Eq. (27), showcasing their accuracy and reliability.

The graphical and tabular representations in this section provide a detailed understanding of the periodic and solitary wave functions described in the study. Figs. 5 through 12 offer visual insights into the spatial, temporal, and intensity characteristics of these wave functions, while Tables 2 through 4 quantitatively assess the accuracy of various analytical and numerical methods. Together, these representations elucidate the physical meanings of the investigated models and validate the employed solution approaches.

## Results and discussion

This study introduces innovative analytical methods, specifically the Khat III, Khat II, and Generalized Rational (GRat) techniques, to derive novel solitary wave solutions for the (2+1)-dimensional graphene sheet (GS) model. The novelty of this research lies in the development and application of these advanced mathematical approaches, which significantly enhance our understanding of the complex dynamics exhibited by graphene. The novelty and scientific contributions of this work are elucidated through the following key outcomes:

1. **Advanced Analytical Methods:** The deployment of the Khat III, Khat II, and GRat methods represents a substantial advancement in the toolbox of analytical techniques for solving nonlinear differential equations. These methods have proven effective in deriving accurate and reliable solitary wave solutions for the (2+1)-dimensional GS model, which is critical for understanding graphene’s dynamic properties.
2. **Improved Accuracy and Validation:** This study rigorously validates the analytical solutions derived using the proposed methods by comparing them with numerical results obtained through the HVI scheme and Adomian decomposition method. This comprehensive validation confirms the high accuracy and reliability of the proposed approaches, underscoring their effectiveness in addressing complex nonlinear models.
3. **Insight into Graphene Behavior:** The solitary wave solutions provide profound insights into the physical phenomena governing the behavior of graphene sheets. These insights are pivotal for the design of advanced materials with tailored properties, which hold significant promise for applications in electronics, photonics, and materials science.
4. **Comprehensive Analysis:** This research conducts an in-depth stability analysis of the derived solutions, supported by detailed graphical representations. The graphical insights demonstrate the robustness of the solutions and provide a clear visualization of the wave characteristics, which are essential for both theoretical advancements and practical applications.
5. **Foundation for Future Research:** The methodologies and findings of this study establish a robust foundation for future investigations in the field. The successful application of the Khat III, Khat II, and GRat methods to the GS model suggests that these techniques could be extended to other complex nonlinear systems, potentially unlocking new directions in applied mathematics and material science.

In conclusion, this study makes significant scientific contributions by introducing novel analytical methods that enhance our understanding of the (2+1)-dimensional GS model. The accurate and reliable solutions derived in this work provide valuable insights into graphene’s behavior, paving the way for future research and technological innovations.

## Conclusion

This study has successfully utilized innovative analytical methods, namely the Khat II, Khat III, and Generalized Rational (GRat) techniques, to derive precise solitary wave solutions for a (2+1)-dimensional graphene sheets (GS) model. These methodologies represent a notable advancement in applied mathematics and material science, offering deeper insights into graphene’s intricate behaviors, which are pivotal for technological applications.

The accuracy of the derived solutions has been thoroughly validated using numerical methods, demonstrating their reliability and real-world applicability. The novel solitary wave solutions presented in this study contribute significantly to the existing literature, providing new perspectives on the dynamics of graphene sheets and paving the way for future research and innovations.

Our approach, which focuses on the idealized dynamics of graphene sheets and excludes external perturbations, emphasizes the fundamental behaviors intrinsic to these materials. While this study is limited to ideal conditions, its findings establish a solid foundation for future research that may address more complex scenarios, including external influences and higher-dimensional models, to better capture the multifaceted nature of graphene.

In conclusion, this study presents a robust framework for understanding the intrinsic properties of graphene sheets through advanced mathematical modeling. The findings have far-reaching implications, potentially aiding the design and development of advanced materials with tailored properties for diverse applications in nanotechnology and materials science.

## Data Availability

The data that support the findings of this study are available from the corresponding author upon reasonable request.
